# NKX2‐5/LHX1 and UHRF1 Establishing a Positive Feedback Regulatory Circuitry Drives Esophageal Squamous Cell Carcinoma through Epigenetic Dysregulation

**DOI:** 10.1002/advs.202413508

**Published:** 2025-04-30

**Authors:** Xukun Li, Dandan Fan, Yong Li, Jian Yuan, Wanyuan Sun, Qinghao Zhu, Ling Qi, Xueling Wu, Jiahui Cai, Tongyang Gong, Ning Zhao, Jianzhong Su, Zhihua Liu, Hongyan Chen

**Affiliations:** ^1^ The State Key Laboratory of Molecular Oncology National Cancer Center/National Clinical Research Center for Cancer/Cancer Hospital Chinese Academy of Medical Sciences and Peking Union Medical College Beijing 100021 P. R. China; ^2^ Shenzhen Key Laboratory of Epigenetics and Precision Medicine for Cancers National Cancer Center/National Clinical Research Center for Cancer/Cancer Hospital & Shenzhen Hospital Chinese Academic of Medical Sciences and Peking Union Medical College Shenzhen 518116 P. R. China; ^3^ Central Laboratory National Cancer Center/National Clinical Research Center for Cancer/Cancer Hospital & Shenzhen Hospital Chinese Academic of Medical Sciences and Peking Union Medical College Shenzhen 518116 P. R. China; ^4^ Oujiang Laboratory, Zhejiang Lab for Regenerative Medicine, Vision and Brain Health, Eye Hospital Wenzhou Medical University Wenzhou Zhejiang 325101 P. R. China; ^5^ Department of Thoracic Surgery, National Cancer Center/National Clinical Research Center for Cancer/Cancer Hospital Chinese Academy of Medical Sciences and Peking Union Medical College Beijing 100021 P. R. China; ^6^ Department of Oncology, National Cancer Center/National Clinical Research Center for Cancer/Cancer Hospital Chinese Academy of Medical Sciences and Peking Union Medical College Beijing 100021 P. R. China

**Keywords:** DNA methylation, LHX1, NKX2‐5, transcriptional dysregulation, UHRF1

## Abstract

DNA methylation regulators play critical roles in modulating oncogenic driver genes in cancers. However, the precise mechanisms through which these DNA methylation regulators influence oncogenesis and clinical therapy have yet to be fully elucidated. This study reveals that hypermethylation of under‐methylated regions (UMRs) within gene bodies is involved in the activation of oncogenic homeobox genes, particularly NKX2‐5 and LHX1, in esophageal squamous cell carcinoma (ESCC). Mechanistically, NKX2‐5 and LHX1 synergistically bind to the promoter region of *UHRF1*, thereby augmenting its transcription. In turn, UHRF1 orchestrates the recruitment of DNMT1/DNMT3A, alongside NKX2‐5 and LHX1, to the UMRs of these genes, thereby increasing DNA methylation levels and their expression. This intricate interplay forms a positive transcriptional feedback loop between NKX2‐5/LHX1 and UHRF1, thus promoting the overexpression of all three genes and ultimately facilitating tumor growth. Notably, concurrent inhibition of UHRF1 and DNMTs impedes tumor growth by suppressing NKX2‐5 and LHX1 expression. Overall, this study identifies a positive feedback regulatory circuitry underlying the UMR hypermethylation‐mediated activation of oncogenic drivers in ESCC and proposes a promising therapeutic strategy for ESCC patients.

## Graphical abstract

The study identifies a positive feed‐forward loop that intertwines DNA methylation regulator UHRF1 with Homeobox genes‐NKX2‐5/LHX1 via UMRs as a critical driver of ESCC. Pharmacological inhibition of UHRF1 and DNMT activity inhibits ESCC growth, which may represent a promising therapeutic approach to treat ESCC patients dependent on NKX2‐5/LHX1/UHRF1 transcription axis for the maintenance of the oncogenic state.

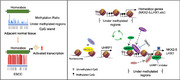

## 1 Introduction

Local DNA hypermethylation and global DNA hypomethylation are prevalent epigenetic alterations observed across various cancer types. The suppression of tumor suppressor genes through local hypermethylation within gene promoter regions is a well‐established phenomenon.^[^ ^1^ ^]^ Conversely, recent studies have revealed a novel link between gene‐body DNA hypermethylation and oncogene activation across multiple cancer types,^[^ ^2^, ^3^ ^]^ thus underscoring the contextual dependence of DNA methylation on gene expression regulation. This complex interplay can lead to the dysregulation of both tumor suppressor genes and oncogenes.

Our research and studies by others have identified numerous conserved and extensive hypomethylated regions (UMRs) in the human genome these regions are characterized by an average methylation level of ≤10% and are enriched with transcriptional regulatory elements, such as promoters and enhancers. UMRs are widely involved in embryonic development and species‐specific phenotypes.^[^ ^4^, ^5^ ^]^ Notably, UMRs also frequently exhibit increased methylation levels in various cancer types, thus indicating their potential role in cancer progression. Functionally, hypermethylation of UMRs may contribute to gene transcription activation by preventing the initiation of intragenic promoters or by modulating the activities of repetitive DNA sequences within the transcriptional unit.^[^ ^6^, ^7^ ^]^

In each cell type, a limited number of transcription factors (TFs) orchestrate a gene regulatory network that is crucial for establishing and maintaining cell identity. These TFs are often termed master regulator TFs. Among them, homeobox genes encode homeoproteins that act as master regulators (MRs), orchestrating morphogenesis, cell proliferation, and cell differentiation. Additionally, homeobox genes play a pivotal role in maintaining cellular identity.^[^ ^8^, ^9^ ^]^ The primary function of homeobox genes is to strike a balance between proliferation and differentiation in normal cells. However, the abnormal expression of homeobox genes can promote oncogenic transformation and tumorigenesis.^[^ ^10^, ^11^ ^]^ Notably, several homeobox genes, such as HOXC10, have been shown to be aberrantly expressed in esophageal squamous cell carcinoma (ESCC).^[^ ^12^ ^]^ Nevertheless, our understanding of the specific roles and mechanisms of homeobox genes in the tumorigenesis of ESCC remains limited. Therefore, it is important to elucidate the characteristics of homeobox genes to enhance our knowledge regarding the specific drivers of ESCC.

In this study, we performed a comprehensive multi‐omics analysis that revealed a novel mechanism whereby gene‐body DNA hypermethylation activates homeobox genes, particularly NKX2‐5 and LHX1, as oncogenic drivers in ESCC. This research revealed a crucial, actionable molecular pathway intertwined with homeobox genes, UHRF1, and the DNA hypermethylation of UMRs, thus playing a pivotal role in promoting ESCC tumorigenesis. The findings of this study may lead to a more effective and targeted treatment option for ESCC.

## 2 Results

### 2.1 Identification of Key Driver Genes in ESCC Based on Multi‐Omics Sequencing Data

**Figure 1:**
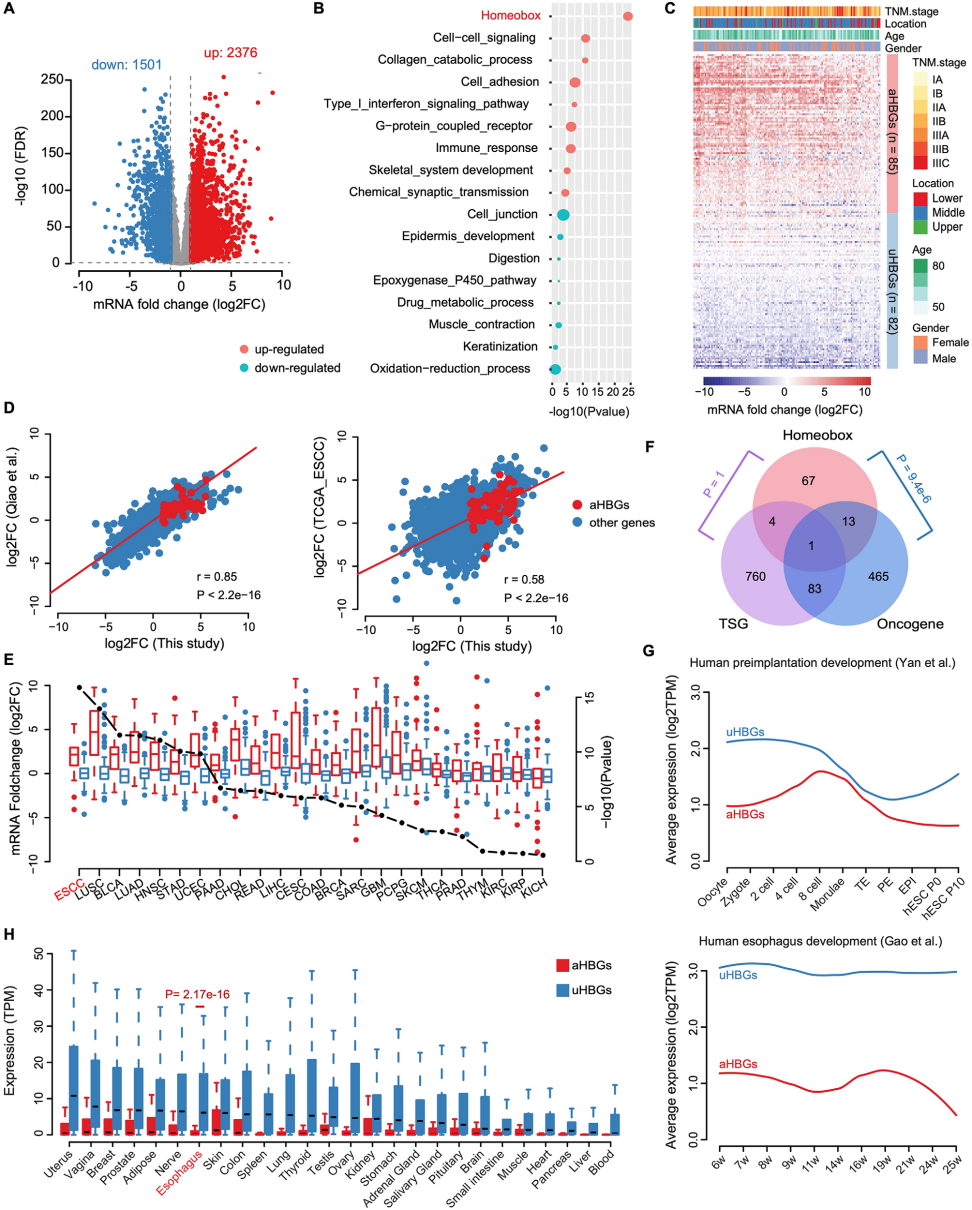
Identification of key driver genes in ESCC based on multi‐omics sequencing data. A) Differentially expressed genes were identified in 150 paired ESCC tissues and matched adjacent normal tissues via RNA‐seq. B) GO enrichment analysis of the differentially expressed genes. The statistical significance of each GO term is presented on the X‐axis, and the enrichment ratio is indicated by the circle size. C) Heatmap of differential homeobox genes in 150 paired ESCC tissues and matched adjacent normal tissues. D) Correlation analysis between the LFC of differential homeobox genes in our dataset and those in the ESCC dataset from Qiao et al. (left) and the TCGA‐ESCA dataset (right). E) Boxplot revealing the expression levels of aHBGs (red) and uHBGs (blue) in multiple TCGA tumor tissues. F) Venn diagram showing the intersection analysis of aHBGs with known oncogenes and tumor suppressor genes. G) Expression levels of aHBGs and uHBGs during the process of human embryo and esophageal development. H) The expression distribution of aHBGs and uHBGs in various mature tissues from GTEx.

To comprehensively elucidate the pathogenesis of ESCC, we conducted comprehensive multi‐omics sequencing data analyses, including Whole Genome sequnecing (WGS), Whole Genome Bisulfite sequencing (WGBS), and Transcriptome sequencing (RNA‐seq), across 150 ESCC samples.^[^ ^13^ ^]^ The RNA‐seq analyses revealed a total of 2376 upregulated and 1501 downregulated genes in ESCC tissues compared with paired adjacent normal tissues (**Figure** **1**A). Subsequently, Gene Ontology (GO) enrichment analysis was performed on these upregulated and downregulated genes and revealed that the upregulated genes were particularly enriched in categories such as “homeobox, cell‒cell signaling, and cell adhesion” (Figure 1B). Notably, the homeobox genes exhibited the highest degree of enrichment. Within this category, we identified 85 homeobox genes that were significantly upregulated and designated them as activated homeobox genes (aHBGs), whereas 82 genes exhibited either downregulation or no change and were labeled as unactivated homeobox genes (uHBGs) in ESCC tissues compared with their normal counterparts (Figure 1C). The upregulation of aHBGs in ESCC tissues was independently confirmed using two publicly available ESCC datasets (Figure 1D). Moreover, The Cancer Genome Atlas (TCGA) pan‐cancer analyses further demonstrated the elevated expression of aHBGs compared with that of uHBGs across multiple tumor types, including ESCC (Figure 1E).

Our investigation further revealed a significantly greater prevalence of oncogenes among aHBGs than would be expected by chance (Figure 1F). Extensive research has underscored the pivotal role of specific spatiotemporal expression patterns of homeobox genes in maintaining the balance between normal cell proliferation and differentiation. Disruption of this pattern has been implicated in cell dysfunction and the facilitation of malignant transformation in normal cells.^[^ ^14^ ^]^ We subsequently investigated the expression patterns of these genes during the process of human embryonic and esophageal development. Both aHBGs and uHBGs exhibited dynamic fluctuations in expression levels, but the expression levels of aHBGs were significantly lower than those of uHBGs throughout this developmental trajectory. Interestingly, aHBG expression decreased to baseline levels during the terminal differentiation of both embryonic and esophageal tissues, thus indicating the near absence of these genes in mature esophageal tissues (Figure 1G). This trend was corroborated by the Genotype‐Tissue Expression (GTEx) portal data, which revealed a much lower aHBG expression than uHBG expression in the majority of differentiated, mature tissues (Figure 1H). Collectively, these findings highlight the overexpression of aHBGs as a salient characteristic in ESCC, suggesting their potential oncogenic function in both the initiation and progression of ESCC.

### 2.2 Hypermethylation of UMRs in the Gene Bodies of aHBGs is Associated with Gene Overexpression

To discern potential distinguishing features between aHBGs and uHBGs, we first performed a systematic genomic analysis utilizing WGS data from 150 ESCC patients. This included gene length, gene compactness, base and codon usage, gene essentiality, and conservation analyses. Our findings revealed that aHBGs presented significantly higher missense Z scores and lower dN/dS ratios than uHBGs (Figure S1A, Supporting Information), which implied that aHBGs are more constrained and intolerant to this class of variations. We further compared the number of single‐nucleotide variants (SNVs) per 1 kb between aHBGs and uHBGs and found that aHBGs presented a much greater frequency of somatic mutations in the TCGA pan‐cancer and ESCC datasets than uHBGs (Figure S1B, Supporting Information). There was a predominance of C>T alterations, which occur predominantly at CpG dinucleotides (Figure S1C,D, Supporting Information). Considering the well‐established link between DNA methylation in CpGs and elevated mutability, especially the greater frequency of CpG‐to‐TpG transitions,^[^ ^15^ ^]^ it is possible that the increased frequency of somatic mutations observed in aHBGs may be linked to their higher methylation status.

**Figure 2:**
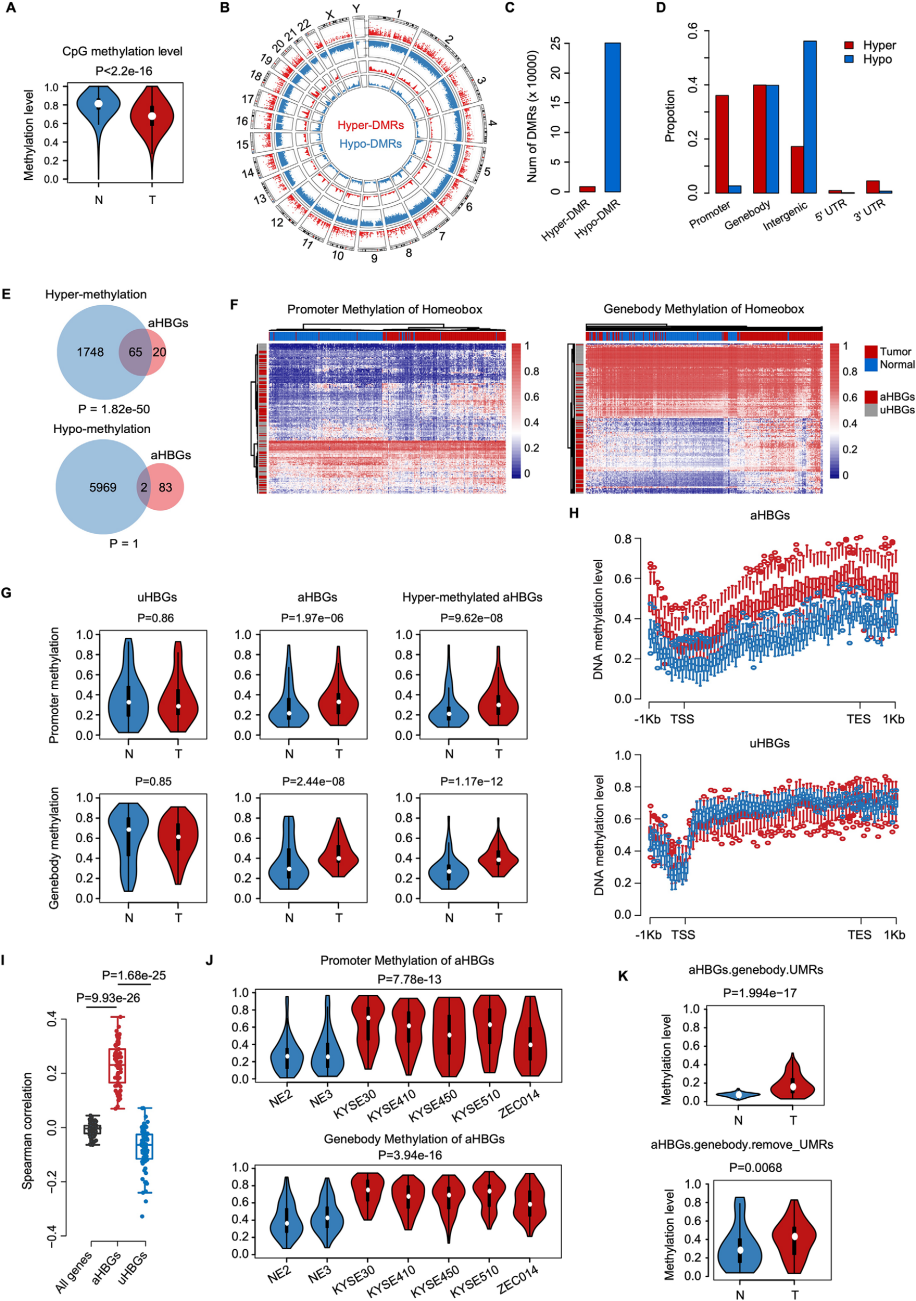
Hypermethylation of UMRs in the gene body regions of aHBGs is positively associated with aHBG overexpression. A) Violin plots with embedded boxplots depict the distribution of average methylation levels at CpG islands in ESCC tissues and paired adjacent normal tissues. B) Circos plot displaying the hyper‐DMRs and hypo‐DMRs identified between ESCC tissues and adjacent normal tissues. C) Numbers of hypermethylated (red) and hypomethylated (blue) DMRs (FDR < 0.05) identified in ESCC tissues. DMRs were defined as regions with a minimum of 10 CpGs and a mean methylation difference ≥0.2. D) Distribution of hyper‐DMRs and hypo‐DMRs across the genome. E) Venn diagram highlighting highly statistically significant overlap between aHBGs and hypermethylated genes. F) Heatmaps of DNA methylation at the promoter (left) and gene body (right) regions of aHBGs and uHBGs. G) Violin plots with embedded boxplots portraying the distribution of average methylation levels at the promoter (upper panel) and gene body (bottom panel) regions of aHBGs and uHBGs in ESCC tissues and adjacent normal tissues. H) The average methylation distribution across the gene bodies of aHBGs and uHBGs in ESCC tissues and adjacent normal tissues. I) Correlation analysis of DNA methylation levels at the gene body regions of aHBGs and uHBGs with their corresponding mRNA levels. J) Violin plots showing the average methylation levels at the promoter (upper) and gene body (bottom) regions of aHBGs in ESCC cells compared with those in normal esophageal epithelial cells. K) Violin plots showing the DNA methylation levels of aHBGs in normal and tumor tissues. The plots represent the methylation levels in two distinct regions: gene body UMRs (upper) of aHBGs, and the remaining gene body regions outside the UMRs (bottom).

To delineate the methylome landscape of esophageal cancer, we conducted a comprehensive analysis of WGBS profiles derived from 150 ESCC tissues and their matched adjacent normal tissues. Our analysis revealed pervasive genome‐wide hypomethylation in ESCC tissues, with a notable decrease in the average CpG methylation level from 81.3% to 67.9% compared with that in normal tissues (**Figure** **2**A; Figure S2A, Supporting Information). By employing a differential methylation threshold of 0.1 and a stringent false discovery rate (FDR) of <0.05 to identify differentially methylated regions (DMRs),^[^ ^16^ ^]^ we identified 8670 hyper‐DMRs and 250696 hypo‐DMRs, indicating an ≈30‐fold enrichment in hypomethylated regions in ESCC tissues compared with normal esophageal tissues. This finding is consistent with the genome‐wide hypomethylation pattern reported by Bivona et al.,^[^ ^17^ ^]^ as 96.6% of DMRs in ESCC tissues exhibited hypomethylation (Figure 2B,C). To gain further insight into these differential methylation patterns, we annotated the DMRs to various genomic regions. Our results revealed that 39.8% of hypo‐DMRs resided within gene bodies, while 56.1% of hypo‐DMRs resided within intergenic regions. The distributions of hyper‐DMRs in ESCC tissues were different, with 36.1% located in promoters, 39.9% within gene bodies, and 17.2% in intergenic regions (Figure 2D). Notably, we observed a hypermethylation phenomenon at aHBGs in ESCC tissues, with 65 out of 85 genes (≈76.5%) exhibiting significant hypermethylation compared with normal tissues (Figure 2E). Notably, the majority of these hypermethylated regions were densely clustered within the gene bodies of aHBGs (Figure 2F–H). Furthermore, spearman correlation analysis was performed to evaluate the relationship between methylation changes (relative to normal tissues) and gene expression changes for three gene sets (all genes, aHBGs, and uHBGs), which revealed a strong positive correlation between DNA hypermethylation within gene body UMRs and the overexpression of aHBGs (Figure 2I). Accordingly, notable variations in methylation patterns were discernible between ESCC cells and normal esophageal epithelial cells (Figure 2J; Figure S2B,C, Supporting Information), thus reinforcing the notion of epigenetic dysregulation in ESCC. Specifically, these differential hypermethylated regions within gene bodies were predominantly located within the UMRs (Figure 2K; Figure S2D, Supporting Information), thereby underscoring the potential functional significance of methylation alterations at these loci. Collectively, these findings suggest that the hypermethylation of UMRs in aHBGs is a pivotal epigenetic hallmark, potentially functioning as a regulatory mechanism that promotes the activation of aHBGs in ESCC.

### 2.3 aHBGs Promoted the Malignant Transformation of Normal Esophageal Epithelial Cells

**Figure 3:**
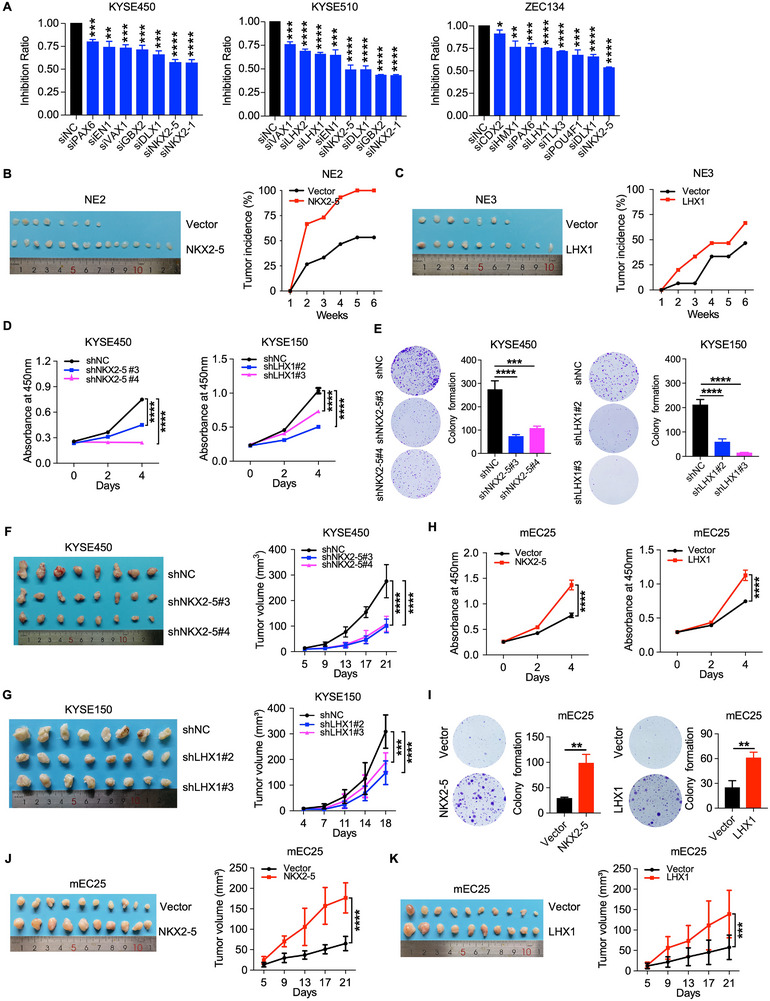
aHBGs promoted the malignant transformation of normal esophageal epithelial cells. A) The rate of proliferation inhibition in ESCC cells was assessed via CCK‐8 assays after candidate aHBGs were knocked down via siRNA. B) NKX2‐5‐overexpressing NE2 cells and control cells were subcutaneously injected into 6‐week‐old female NCG mice. Representative tumor images (left) and tumor incidence data (right) are shown (n = 15). C) LHX1‐overexpressing NE3 cells and control cells were subcutaneously injected into 6‐week‐old female NCG mice. Representative tumor images (left) and tumor incidence data (right) are shown (n = 15). D,E) CCK‐8 and colony formation assays were performed after the knockdown of NKX2‐5 or LHX1 in KYSE450 and KYSE150 cells. Representative images of colonies (left) and statistical analyses of the number of colonies (right) are shown. F,G) Representative tumor images (left) and tumor growth curves (right) of mice subcutaneously injected with NKX2‐5‐knockdown KYSE450, LHX1‐knockdown KYSE150, and control cells (n = 8). H,I) CCK‐8 and colony formation assays were performed after the overexpression of NKX2‐5 or LHX1 in mEC25 cells. Representative images of colonies (left) and statistical analyses of the number of colonies (right) are shown. J,K) Representative tumor images (left) and tumor growth curves (right) of mice subcutaneously injected with NKX2‐5‐ and LHX1‐overexpressing mEC25 cells and control cells (n = 10). The data are presented as the means ± SD; two‐tailed *t*‐tests, **P < 0.01, ***P < 0.001, ****P < 0.0001.

To define the biological functions of aHBGs in ESCC tumorigenesis, we performed a series of in vitro and in vivo gain‐ or loss‐of‐function experiments. By integrating multiple molecular features, we constructed a gene risk score (GRS) system (Section 4.5) to assess the risk of aHBGs. Based on the GRS ranking, we performed Gene Set Enrichment Analysis (GSEA) to identify associated functional pathways. From the top 15 GRS‐ranked genes, we further selected 13 candidate aHBGs for validation based on their functional enrichment in cancer‐related pathways. These 13 candidate genes, including PAX6, NKX2‐5, NKX2‐1, LHX2, LHX1, DLX1, TLX3, CDX2, HMX1, GBX2, POU4F1, VAX1, and EN1 (Figure S3A–C, Supporting Information). Subsequently, we employed small interfering RNAs (siRNAs) to knockdown these aHBGs and observed significant suppression of ESCC cell proliferation, with NKX2‐5, LHX1, DLX1, GBX2, and TLX3 exhibiting the most pronounced inhibitory effects (**Figure** **3**A). To further substantiate their oncogenic potential, we generated stable cells overexpressing these five candidates in normal esophageal epithelial cells (NE2 or NE3) and subjected them to malignant transformation assays in vivo (Figure S3D, Supporting Information). By subcutaneously implanting these cells into NCG mice, we monitored their tumorigenic capacity. Evidently, the tumorigenic rates of mice injected with NKX2‐5‐ and LHX1‐overexpressing cells were relatively higher than those of their respective control groups (Figure 3B,C). Furthermore, sphere formation assays confirmed that the overexpression of NKX2‐5 and LHX1 significantly enhanced the sphere formation capability (Figure S3E,F, Supporting Information). Collectively, our findings underscore the critical roles of NKX2‐5 and LHX1 as essential drivers of the malignant transformation of normal esophageal epithelial cells.

To further characterize the oncogenic functions of NKX2‐5 and LHX1 in ESCC, we constructed several stable ESCC cell lines featuring either knockdown or overexpression of these two genes (Figure S4A–D, Supporting Information). We used CCK‐8 assays, colony formation studies, and tumor transplantation models to quantitatively assess the impacts of NKX2‐5 and LHX1 on ESCC cell proliferation. Our findings revealed that silencing NKX2‐5 or LHX1 significantly inhibited ESCC cell proliferation, colony formation, and tumor growth (Figure 3D–G; Figure S4E–H, Supporting Information). Conversely, the overexpression of either NKX2‐5 or LHX1 stimulated cell growth both in vitro and in vivo (Figure 3H–K; Figure S4I,J, Supporting Information). Collectively, these comprehensive results reinforce the pivotal status of NKX2‐5 and LHX1 as essential drivers of ESCC growth.

### 2.4 NKX2‐5 and LHX1 Cooperatively Activated the Expression of UHRF1

**Figure 4:**
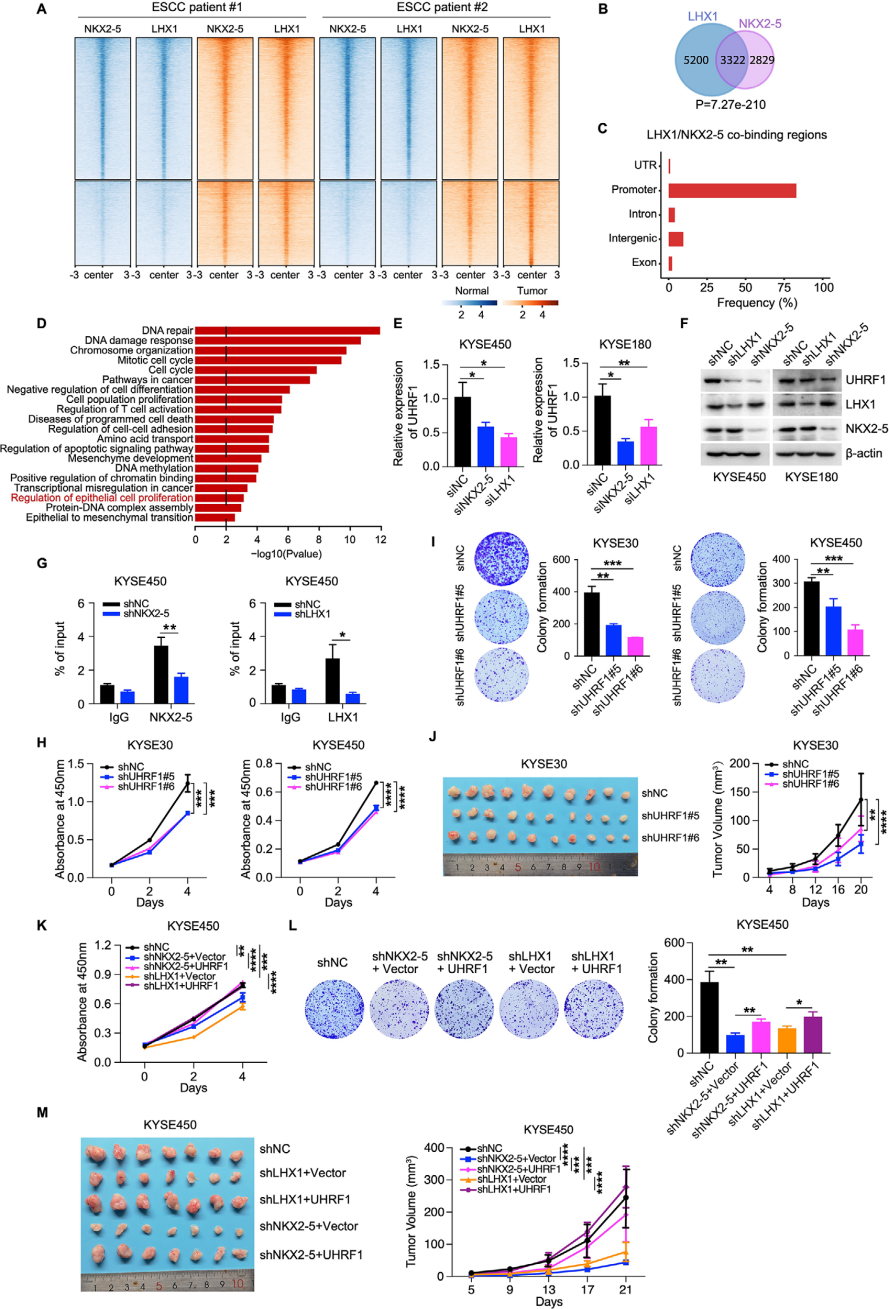
NKX2‐5 and LHX1 transcriptionally regulate the expression of UHRF1. A) Heatmap showing the NKX2‐5 and LHX1 ChIP‐seq signal intensities in two samples of ESCC tissues and paired adjacent normal tissues. The color bars at the bottom represent the number of reads per million normalized signals. B) Venn diagram displaying the overlapping binding peaks of the NKX2‐5 and LHX1 ChIP‐seq signals in ESCC tissues. C) Distribution of NKX2‐5 and LHX1 co‐binding sites in ESCC tissues according to ChIP‐seq analysis. D) GO functional enrichment analysis of genes coregulated by NKX2‐5 and LHX1; the enriched GO terms were ranked by −log10*P*. E) qRT‒PCR analysis of UHRF1 expression in KYSE450 and KYSE180 cells transfected with NKX2‐5, LHX1 siRNA, or control siRNA. F) The protein level of UHRF1 in KYSE450 and KYSE180 cells infected with NKX2‐5 or LHX1 shRNA lentivirus or control lentivirus. G) ChIP‒qPCR assays showing the changes in the binding of NKX2‐5 and LHX1 at the promoter region of the *UHRF1* locus between NKX2‐5‐ and LHX1‐knockdown KYSE450 cells and control cells. The binding sites of NKX2‐5 and LHX1 on the promoter of *UHRF1* located at chr19:4911559‐4911883 (NKX2‐5) and chr19:4911375‐4911880 (LHX1). H) CCK‐8 assays of the proliferation of UHRF1‐knockdown KYSE30 and KYSE450 cells and control cells. I) Colony formation assays (left) and statistical analyses (right) of UHRF1‐knockdown KYSE30, KYSE450, and control cells. J) Representative tumor images (left) and tumor growth curves (right) of mice subcutaneously injected with UHRF1‐knockdown KYSE30 cells and control cells (n = 10). K,L) CCK‐8 and colony formation assays with exogenous overexpression of UHRF1 in NKX2‐5‐ and LHX1‐knockdown KYSE450 cells. Representative images of colonies (left) and statistical analyses of the number of colonies (right) are shown. M) Representative tumor images (left) and tumor growth curves (right) of mice subcutaneously injected with LHX1‐ and NKX2‐5‐knockdown KYSE450 cells plus the overexpression of UHRF1 and control cells (n = 7). The data are presented as the means ± SD; two‐tailed *t*‐tests, *P < 0.05, **P < 0.01, ***P < 0.001, ****P < 0.0001.

To elucidate the molecular mechanisms underlying NKX2‐5/LHX1‐driven ESCC growth, we first performed ChIP‐seq to define the NKX2‐5/LHX1 cistromes in ESCC tissues and their adjacent normal counterparts. Although the overall binding patterns are similar between normal and tumor tissues, k‐means clustering identified two main categories of binding patterns: one that is enriched in both normal and tumor tissues, and another that is significantly enriched only in tumor tissues. Thus, compared to normal tissues, tumor tissues show greater overall enrichment of NKX2‐5 and LHX1 binding (**Figure** **4**A). Notably, the genomic distribution of these binding sites exhibited a considerable overlap, with a statistical significance of *P* = 7.27e‐210 (Figure 4B), indicating that approximately 54% of the NKX2‐5 binding peaks coincided with LHX1 binding peaks. Further analysis revealed that both transcription factors predominantly colocalized within promoter regions (Figure 4C; Figure S5A, Supporting Information), suggesting a concerted regulatory mechanism.

To identify the downstream effectors of this collaborative regulation, we integrated the NKX2‐5/LHX1 cistromes with the differential gene expression profiles derived from 150 ESCC cases. GO term enrichment analyses revealed that genes coregulated by NKX2‐5 and LHX1 were involved in DNA repair, the DNA damage response, the mitotic cell cycle, cell differentiation, the apoptotic signaling pathway, cell–cell adhesion, chromatin binding, the epithelial–mesenchymal transition, and epithelial cell proliferation (Figure 4D). Thus, these findings underscore the extensive influence of NKX2‐5 and LHX1, as they cooperatively occupy regulatory regions of key genes within cancer‐pertinent pathways on a genome‐wide scale.

To identify the putative functional target genes coregulated by NKX2‐5 and LHX1, we focused on the “epithelial cell proliferation” term and measured the expression of these genes following the depletion of NKX2‐5 and LHX1. Interestingly, ubiquitin‐like protein with plant homeodomain and ring finger domains 1 (UHRF1) emerged as a compelling target due to its critical role in tumorigenesis and potential as an attractive drug target for treatment.^[^ ^18^, ^19^ ^]^ Subsequent RT‐qPCR and Western blot results revealed a significant downregulation of UHRF1 expression in ESCC cells upon NKX2‐5 and LHX1 silencing (Figure 4E,F; Figure S5B,C, Supporting Information). Further analysis revealed that both NKX2‐5 and LHX1 co‐occupied the promoter region of *UHRF1*, accompanied by elevated levels of active histone markers such as H3K4me3, H3K4me1, and H3K27ac, indicative of active transcription (Figure S5D, Supporting Information).

To confirm the direct binding of NKX2‐5 and LHX1 to the *UHRF1* promoter, we conducted ChIP assays, which specifically targeted the genomic region spanning from 1 to 1.5 kb upstream of the transcription start site (TSS) of *UHRF1*. The binding of NKX2‐5 and LHX1 relative to the IgG control was significantly enriched in the promoter region of the *UHRF1* locus, which was decreased by NKX2‐5 and LHX1 ablation (Figure 4G; Figure S5E, Supporting Information). Taken together, these results indicate that NKX2‐5 and LHX1 co‐bound and cooperatively activated the expression of UHRF1.

Previous studies have firmly established the robust correlation between UHRF1 overexpression and tumorigenesis,^[^ ^20^, ^21^ ^]^ prompting us to delve into the biological function of this association in ESCC. As anticipated, downregulation of UHRF1 impeded the proliferation, colony formation, and tumorigenic potential of ESCC cells (Figure 4H–J; Figure S5F–H, Supporting Information). To assess the precise contribution of UHRF1 to NKX2‐5/LHX1‐induced ESCC growth, we restored UHRF1 expression in NKX2‐5‐ and LHX1‐depleted ESCC cells and performed cell growth assays. Notably, the inhibitory effects on growth caused by NKX2‐5 and LHX1 depletion were alleviated by restoring UHRF1 expression both in vitro and in vivo (Figure 4K–M; Figure S5I–K, Supporting Information). Collectively, these results support that NKX2‐5 and LHX1 drive ESCC growth by increasing the expression of UHRF1.

### 2.5 UHRF1‐Mediated DNA Hypermethylation of Gene Bodies Induced the Overexpression of NKX2‐5 and LHX1

**Figure 5:**
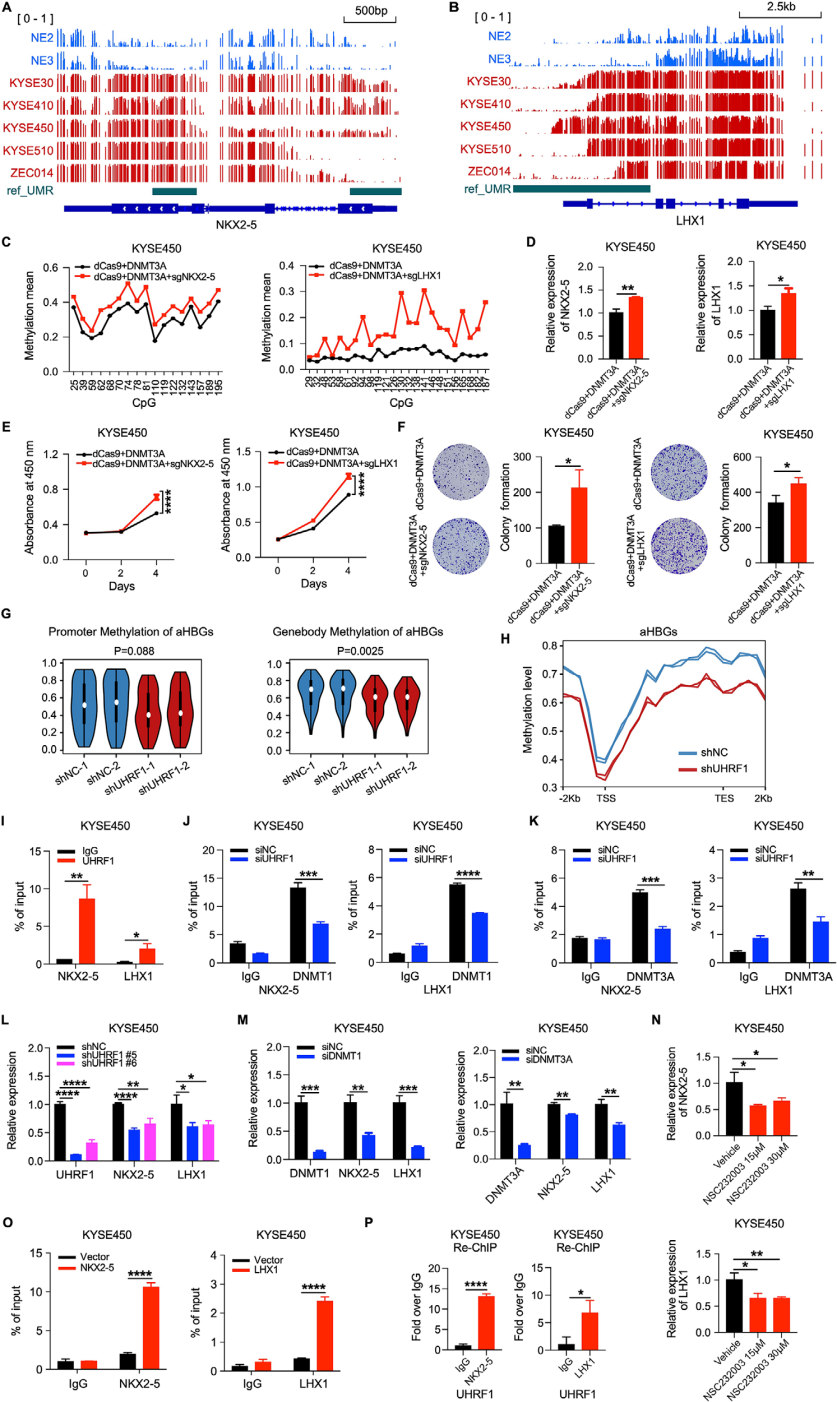
DNA hypermethylation induced the overexpression of oncogenic homeobox genes. A,B) Integrative Genomic Viewer (IGV) displaying the DNA methylation patterns at the gene bodies of the *NKX2‐5* and *LHX1* loci in normal esophageal epithelial cells and ESCC cells. Specifically, the NKX2‐5 UMR is located at chr5: 173232961–173233309 and chr5: 173234864–173235362, and the LHX1 UMR is located at chr17: 36937074–36940522. C,D) The DNA methylation levels at the gene‐body UMRs of the *NKX2‐5* and *LHX1* loci and NKX2‐5 and LHX1 expression were examined in KYSE450 cells treated with or without DNA methylation editing systems. E,F) CCK‐8 and colony formation assays of KYSE450 cells treated with or without DNA methylation editing systems. Representative images of colonies (left) and statistical analyses of the number of colonies (right) are shown. G) Violin plots showing the average methylation levels at the promoter (left) and gene body (right) regions of aHBGs in UHRF1‐knockdown KYSE450 cells and control cells. H) DNA methylation distribution across the gene bodies of aHBGs in UHRF1‐knockdown KYSE450 cells and control cells. I) ChIP‒qPCR assays showing the binding of UHRF1 at the UMRs of the *NKX2‐5* and *LHX1* loci in KYSE450 cells. The binding sites of UHRF1 on the UMR of *NKX2‐5* and *LHX1* located at chr5: 173234860–173235206 (NKX2‐5) and chr17: 36937475–36940110 (LHX1). J,K) KYSE450 cells were transfected with UHRF1 siRNA or control siRNA for 60 h. Then, ChIP assays were performed with DNMT1 and DNMT3A antibodies, followed by qPCR to detect changes in the binding of DNMT1 and DNMT3A at the UMRs of the *NKX2‐5* and *LHX1* loci. The binding sites of DNMT1, DNMT3A on the UMR of *NKX2‐5* and *LHX1* located at chr5: 173234860–173235206 (NKX2‐5) and chr17: 36937475–36940110 (LHX1). L) qRT‒PCR analysis of UHRF1, NKX2‐5, and LHX1 expression in KYSE450 cells infected with UHRF1 shRNA lentivirus or control lentivirus. M) qRT‒PCR analysis of DNMT1, DNMT3A, NKX2‐5, and LHX1 expression in KYSE450 cells transfected with DNMT1, DNMT3A siRNA, or control siRNA. N) qRT‒PCR analysis of NKX2‐5 and LHX1 expression in KYSE450 cells treated with the UHRF1 inhibitor NSC232003 or vehicle. O) KYSE450 cells were transfected with pLVX‐puro‐NKX2‐5/LHX1 plasmids or control plasmids for 60 h. Then, ChIP‒qPCR assays were performed to examine the binding of NKX2‐5/LHX1 at the UMRs of the *NKX2‐5*/*LHX1* loci. The binding sites of NKX2‐5 and LHX1 on the UMR of *NKX2‐5* or *LHX1* located at chr5:173234860‐173235206 (NKX2‐5) and chr17:36937475‐36940110 (LHX1). P) Re‐ChIP analysis was performed to verify the co‐occupancy of NKX2‐5/UHRF1 and LHX1/UHRF1 at the UMRs of the *NKX2‐5* and *LHX1* loci, respectively. The binding sites of UHRF1, NKX2‐5, and LHX1 on the UMR of *NKX2‐5* and *LHX1* located at chr5:173234860‐173235206 (NKX2‐5) and chr17:36937475‐36940110 (LHX1). The data are presented as the means ± SD; two‐tailed *t*‐tests, *P < 0.05, **P < 0.01, ***P < 0.001, ****P < 0.0001.

To better understand the precise mechanism underlying NKX2‐5 and LHX1 overexpression in ESCC, we conducted a comprehensive analysis of WGBS data from ESCC cells and normal esophageal epithelial cells. Our findings revealed a notable hypermethylation pattern in the gene‐body UMRs of the *NKX2‐5* and *LHX1* loci in ESCC cells, mirroring the alterations observed in ESCC tissues (**Figure** **5**A,B; Figure S6A, Supporting Information). To validate the pivotal role of DNA hypermethylation in driving the overexpression of NKX2‐5 and LHX1, we employed the De‐activated Cas9 (dCas9)‐DNMT3A‐DNA methylation editing systems, which dCas9 was fused to DNMT3A to specifically methylate the UMRs within the *NKX2‐5* and *LHX1* loci mediated by sgRNA. Subsequent bisulfite sequencing confirmed a significant elevation in DNA methylation levels at these gene‐body UMRs in ESCC cells (Figure 5C). Concurrent with this increase in DNA methylation, we observed an obvious increase in the expression of *NKX2‐5* and *LHX1* (Figure 5D). Accordingly, the DNMT3A‐induced methylation of the *NKX2‐5* and *LHX1* loci accelerated cell growth (Figure 5E,F). Collectively, these findings underscore the critical importance of enhanced DNA methylation within gene‐body UMRs as a key mechanism that activates the expression of *NKX2‐5* and *LHX1*, thereby promoting the growth of ESCC.

Given the pivotal role of UHRF1 in establishing and maintaining DNA methylation patterns in mammalian cells,^[^ ^18^ ^]^ we next investigated whether UHRF1 is essential for DNA methylation in the UMRs of homeobox genes. Our WGBS analyses revealed a significant reduction in DNA methylation levels at the gene body regions of aHBGs, particularly *NKX2‐5* and *LHX1*, upon UHRF1 knockdown (Figure 5G,H; Figure S6B,C, Supporting Information). To further substantiate this finding, we conducted UHRF1 ChIP assays, which confirmed the enriched binding of UHRF1 at the UMRs of the *NKX2‐5* and *LHX1* loci compared with the IgG control (Figure 5I).

Given that UHRF1 orchestrates DNA methylation by recruiting DNMTs to chromatin,^[^ ^18^ ^]^ we next examined the interaction between UHRF1 and DNMT1/DNMT3A. Immunoprecipitation (IP) assays revealed a physical interaction between UHRF1 and these DNMTs, which was disrupted upon UHRF1 knockdown (Figure S6D, Supporting Information). To determine whether this interaction occurs on chromatin, we performed DNMT1/DNMT3A ChIP assays following UHRF1 ablation. Notably, the recruitment of DNMT1/DNMT3A to the UMRs of the *NKX2‐5* and *LHX1* loci was markedly downregulated upon UHRF1 depletion (Figure 5J,K; Figure S6E, Supporting Information). These findings underscore the critical role of UHRF1 in facilitating DNA methylation at the UMRs of homeobox genes through its interaction with DNMT1/DNMT3A. To characterize the UHRF1‐mediated transcriptional activation of *NKX2‐5* and *LHX1*, we conducted expression analyses in KYSE450 and KYSE510 cells after UHRF1/DNMT1/DNMT3A silencing. Notably, the ablation of these factors led to marked suppression of *NKX2‐5* and *LHX1* expression (Figure 5L,M; Figure S6F–H, Supporting Information). Similarly, treatment with the UHRF1 inhibitor NSC232003 also significantly repressed the expression of both genes (Figure 5N; Figure S6I, Supporting Information). Interestingly, we found that NKX2‐5 and LHX1 can bind to their respective UMRs, thereby activating their own gene expression (Figure 5O; Figure S6J, Supporting Information). To further elucidate the role of UHRF1 in regulating the transcription of *NKX2‐5* and *LHX1*, we performed IP and sequential ChIP (Re‐ChIP) assays. IP assay demonstrated a physical interaction between UHRF1 and both NKX2‐5 and LHX. Furthermore, the Re‐ChIP assay revealed the formation of a regulatory complex encompassing UHRF1 and both NKX2‐5 and LHX1 at their respective genomic loci (Figure 5P; Figure S6K, Supporting Information). Together, these results suggest that UHRF1 orchestrates the recruitment of DNMT1/DNMT3A, alongside NKX2‐5 and LHX1, to the UMRs of these genes, thereby increasing their expression.

### 2.6 Therapeutic Potential of the NKX2‐5/LHX1/UHRF1 Feedback Loop in Primary ESCC

**Figure 6:**
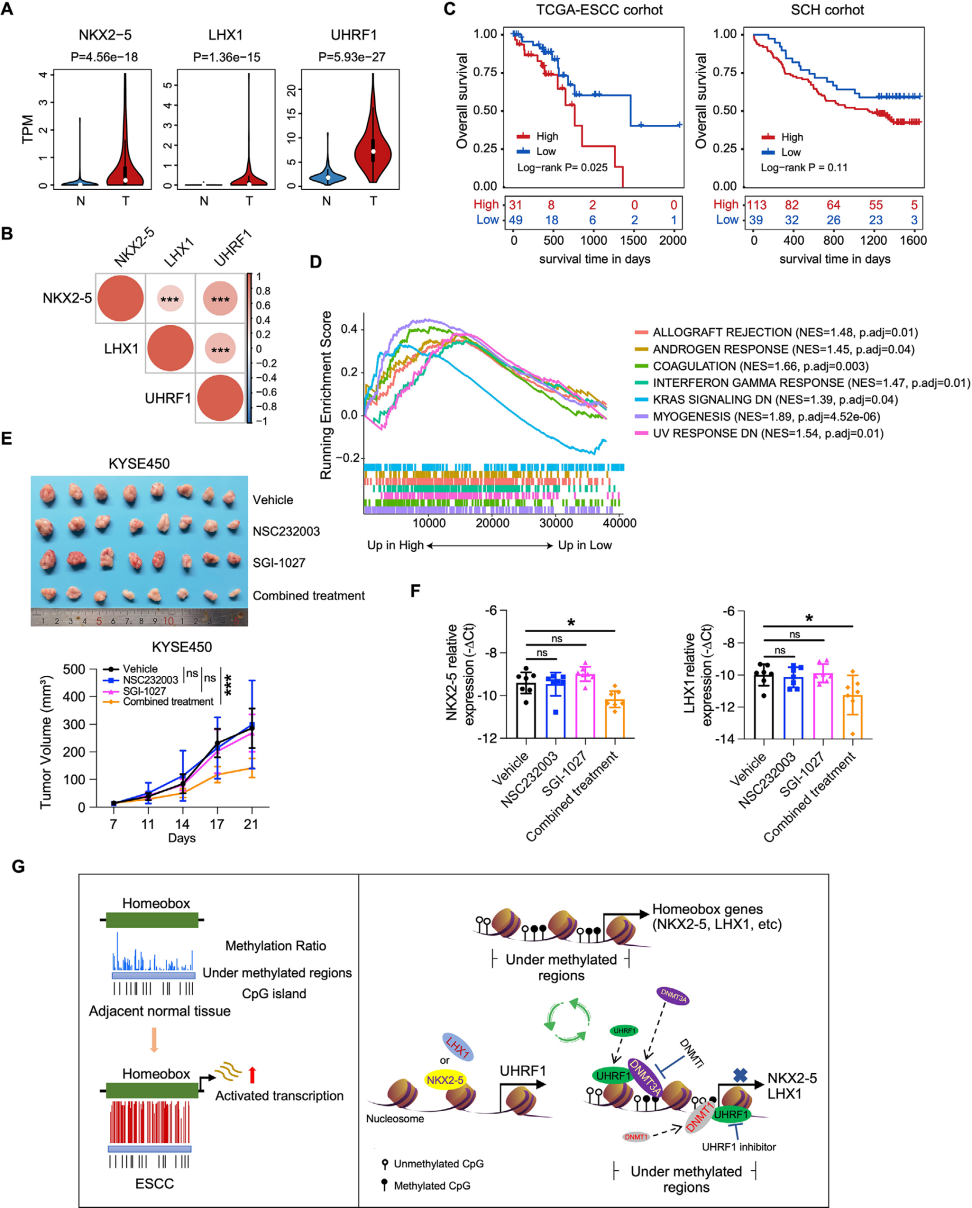
Prognostic and therapeutic potential of NKX2‐5/LHX1/UHRF1 in ESCC. A) The expression of *NKX2‐5*, *LHX1*, and *UHRF1* was analyzed in 150 ESCC tissues and paired adjacent normal tissues. B) The correlation between the expression of UHRF1 and the expression of NKX2‐5 and LHX1 in ESCC tissues. C) Kaplan‒Meier analysis of the overall survival of ESCC patients in the TCGA and SCH cohorts. ESCC patients were divided into two groups according to the pathway activity score of the regulatory axis comprising NKX2‐5, LHX1, UHRF1, DNMT1, and DNMT3A. The log‐rank test *p‐*values are shown. D) Pathway analysis of differentially expressed genes in the high‐activity score groups on the regulatory axis. E) Representative tumor images (upper) and tumor growth curves (bottom) of mice injected with KYSE450 cells treated with the UHRF1 inhibitor, DNMT inhibitor, combined treatment, or vehicle (n = 7). F) qRT‒PCR analysis of NKX2‐5 and LHX1 expression in tumor tissues derived from mice treated with the UHRF1 inhibitor, DNMT inhibitor, combined treatment, or vehicle. G) Mechanistic diagram of the positive‐feedback regulatory loop between NKX2‐5/LHX1 and UHRF1 in ESCC. The data are presented as the means ± SD; two‐tailed *t*‐tests, *P < 0.05, ***P < 0.001; ns means no significance.

To validate the existence of a feedback loop involving NKX2‐5, LHX1, and UHRF1 in a clinical context, we analyzed 150 paired ESCC clinical samples. Our findings revealed a concerted upregulation of these three genes in ESCC tissues compared with their adjacent normal counterparts (**Figure** **6**A). Furthermore, a positive correlation was observed between the expression levels of *NKX2‐5* or *LHX1* and *UHRF1* in these clinical ESCC samples (Figure 6B; Figure S7A, Supporting Information). These data support the clinical relevance of the NKX2‐5/LHX1/UHRF1 feedback loop in ESCC.

To investigate the prognostic implications of the NKX2‐5/LHX1/UHRF1 axis in ESCC patients, we constructed a comprehensive regulatory model incorporating NKX2‐5, LHX1, UHRF1, DNMT1, and DNMT3A. We subsequently calculated a patient‐level pathway activity score for this regulatory axis via the PLAGE method from the GSVA package. The Maxstat package was then used to calculate the optimal cutoff value based on GSVA scores, and patients were divided into high‐ and low‐activity groups accordingly. Kaplan‐Meier survival analysis was performed to assess the prognostic impact of this stratification. Notably, patients in the low‐activity group had significantly better survival outcomes than those in the high‐activity group in the TCGA‐ESCC cohort (log‐rank *P* = 0.025). While our SCH cohort did not reach statistical significance in terms of survival difference (log‐rank *P* = 0.11), a trend toward inferior survival was still apparent in the high‐activity group compared with the low‐activity group (Figure 6C), further corroborating the prognostic value of this regulatory axis in ESCC. Furthermore, pathway analysis revealed that DEGs in the high‐activity group were enriched in functions related to immunosuppressive responses or immune evasion processes, such as allograft rejection and interferon gamma response (Figure 6D), thus suggesting that the high‐activity may promote ESCC tumorigenesis and progression by mediating the interaction between tumor cell and tumor microenvironment.

These findings prompted us to explore the therapeutic potential of combining UHRF1 inhibition with DNMT blockade for ESCC growth suppression. Specifically, KYSE450 cells, which are characterized by high NKX2‐5/LHX1/UHRF1 expression, were injected subcutaneously into BALB/c nude mice. One week later, the mice were randomly divided into four groups and administered either the UHRF1 inhibitor NSC232003, the DNMT inhibitor SGI‐1027, the combination of NSC232003 and SGI‐1027 or the vehicle control weekly. Our results demonstrated that combined treatment with NSC232003 and SGI‐1027 led to a more pronounced downregulation of NKX2‐5/LHX1 expression and superior tumor growth inhibition than either inhibitor alone (Figure 6E,F; Figure S7B, Supporting Information), highlighting the potential of this combinatorial therapeutic approach for ESCC management. Collectively, our findings reveal the presence of a positive feedback regulatory circuit between NKX2‐5/LHX1 and UHRF in ESCC. The concurrent inhibition of UHRF1 and DNMTs significantly enhanced therapeutic responsiveness, thus revealing a promising therapeutic strategy for patients with ESCC exhibiting NKX2‐5/LHX1/UHRF1 overexpression (Figure 6G).

## 3 Discussion

In this study, we delineated the distinct transcriptome alterations that distinguish ESCC from normal esophageal epithelial tissues, and the upregulation of oncogenic homeobox genes was identified as a prominent hallmark. Furthermore, we performed a comprehensive methylation analysis and revealed a pervasive hypomethylation landscape in ESCC, juxtaposed with aberrant hypermethylation of CGI promoters and UMRs within gene bodies. Interestingly, it was observed that the hypermethylation of UMRs, rather than promoter regions, was linked to the active gene transcription of aHBGs. However, no correlation was found between the hypermethylation of UMRs and the transcription of uHBGs. Although previous studies have identified DMRs in ESCC, tumor‐specific hyper‐DMRs were not found to be enriched in cancer‐related pathways.^[^ ^22^ ^]^ Furthermore, a previous study primarily focused on the inverse relationship between DNA methylation and gene expression in ESCC.^[^ ^23^ ^]^ Our study is the first to identify a positive correlation between the hypermethylation of UMRs and potentially oncogenic homeobox genes, thus highlighting the importance of DNA hypermethylation in regulating oncogenic genes in ESCC.

Homeobox genes exhibit abnormal expression across many types of cancer.^[^ ^24^, ^25^, ^26^ ^]^ However, a systemic investigation of their expression patterns in ESCC has been lacking. In this study, we revealed that upregulated homeobox genes in ESCC are typically expressed during esophageal development and/or undifferentiated cells, whereas downregulated homeobox genes are characteristic of adulthood and/or differentiated tissues. This finding is consistent with the general pattern of homeobox gene dysregulation observed in cancer.^[^ ^14^ ^]^

To determine the direct functional roles of homeobox genes in ESCC, we focused on the 85 homeobox genes upregulated by the hypermethylation of UMRs, as these genes constituted the most strongly enriched category in ESCC. Notably, two of these genes, NKX2‐5 and LHX1, were found to play pivotal roles in the oncogenic transformation of normal esophageal epithelial cells and ESCC tumor growth. These findings also underscored the critical roles of these genes in initiation of esophageal tumorigenesis. The overexpression of LHX1, which is a member of the LIM family of homeodomain proteins, can lead to oncogenic activity, thereby augmenting malignant cellular behaviors.^[^ ^27^ ^]^ Conversely, NKX2‐5 serves as a tumor suppressor in the context of colorectal cancer.^[^ ^28^ ^]^ However, both LHX1 and NKX2‐5 exhibit oncogenic properties in ESCC, thus underscoring the intricate and context‐specific nature of homeobox gene functionality in cancer development.

The observed correlation between the hypermethylation of UMRs and aHBGs prompted us to further verify the underlying regulatory mechanism in cell models. Like the patterns observed in tissue samples, we found that the gene body UMRs residing at the *NKX2‐5* and *LHX1* loci were notably hypermethylated in ESCC cells compared with normal esophageal epithelial cells. Convincingly, our dCas9‐DNMT3A‐mediated methylation editing experiments confirmed that the hypermethylation of these UMRs can directly upregulate NKX2‐5 and LHX1 expression, thereby promoting ESCC cell proliferation. These findings underscore the importance of UMR hypermethylation as a crucial epigenetic mechanism that is responsible for activating the oncogenic potential of NKX2‐5 and LHX1 in ESCC.

Notably, our investigation revealed a fascinating interplay between NKX2‐5 and LHX1, as they collaborated to govern a transcriptional network. These two factors converge on regulatory regions across the genome, cooperatively modulating the expression of genes pivotal for epithelial cell proliferation, differentiation, etc. Notably, we confirmed that NKX2‐5 and LHX1 jointly occupy the promoter region of *UHRF1*, thereby activating its transcription. Importantly, functional rescue experiments revealed the pivotal role of NKX2‐5 and LHX1 in promoting ESCC cell growth via the oncogenic function of UHRF1. Thus, our findings revealed a novel molecular mechanism whereby UHRF1 was coregulated by NKX2‐5 and LHX1, potentially contributing to the proliferation of ESCC cells.

Although our previous study established that DNMT3A sustains the methylation of UMRs,^[^ ^4^ ^]^ the epigenetic regulators responsible for recruiting DNMT3A to these regions have not been revealed. UHRF1 is a pivotal regulator of DNA methylation, and it engages in complex interactions with epigenetic modulators such as DNMT1, DNMT3A/B, HDAC1, and PRMT5 to facilitate DNA methylation maintenance and histone modifications that govern gene expression.^[^ ^18^, ^29^, ^30^ ^]^ Therefore, we hypothesized that UHRF1 might bind to UMRs, thereby increasing DNA methylation levels and consequently activating *NKX2‐5* and *LHX1* transcription in ESCC cells. Indeed, WGBS analyses revealed that UHRF1 depletion specifically diminished the DNA methylation levels within the aHBG gene body, and ChIP assays corroborated that UHRF1 directly bound to the methylated UMRs residing at the *NKX2‐5* and *LHX1* loci. Furthermore, we demonstrated that UHRF1 interacts with DNMT1/DNMT3A, orchestrating their recruitment to UMRs located at the *NKX2‐5* and *LHX1* loci, thereby transcriptionally activating the expression of these genes. Consequently, alterations in UMR methylation pave the way for the establishment of a feed‐forward loop. UHRF1 binds to hypermethylated UMRs, thus triggering the transcription of *NKX2‐5* and *LHX1*. In turn, the elevated levels of NKX2‐5 and LHX1 further increase UHRF1 transcription, reinforcing the occupancy of UHRF1 at the UMRs of *NKX2‐5* and *LHX1* to sustain their transcription.

Overall, we pinpointed the pivotal regulator of DNA methylation within UMRs and elucidated a plausible mechanism by which this process modulates the expression of homeobox genes. Notably, these two homeobox genes exhibit an intricate autoregulatory feedback loop, which is reinforced by their binding to their respective UMRs, thereby increasing their own expression. This finding is consistent with the “core transcription regulatory circuitry (CRC)” paradigm that is prevalent across diverse cell types.^[^ ^31^, ^32^ ^]^ More importantly, inhibiting UHRF1 in conjunction with disrupting DNA methylation activity effectively dampened the epigenetically activated UHRF1/NKX2‐5/LHX1 feedback loop, thereby inhibiting ESCC growth. While targeting either UHRF1 or DNMTs individually is inadequate for suppressing the expression of NKX2‐5/LHX1, indicating that the UHRF1‐mediated recruitment of DNMTs is crucial for NKX2‐5/LHX1 transcription. As a result, monotherapy failed to exhibit anti‐tumor efficacy, emphasizing the importance of disrupting the regulatory axis in a synergistic manner. Additionally, these findings further validate that simultaneous inhibition of both UHRF1 and DNMTs exhibits anti‐tumor activity by effectively suppressing the expression of NKX2‐5/LHX1. In line with the notion that gene body methylation represents a viable therapeutic target in cancer,^[^ ^33^ ^]^ our results provide a novel epigenetic treatment strategy that specifically targets gene body DNA methylation in ESCC.

However, it has several limitations. Firstly, while our current evidence offers mechanistic insights into how this feedback loop sustains tumor growth, the initial establishment of this loop still poses an enigma that requires further exploration. Secondly, potential cofactors that could influence the expression of homeobox genes due to DNA hypermethylation remain unidentified, necessitating further research in the future. Thirdly, our study primarily focuses on tumor cells, and thus, the impact on the tumor microenvironment and the underlying mechanism of our identified epigenetic regulatory axis requires further investigation.

Taken together, a fundamental aspect of the core regulatory circuitry model was discovered, illustrating a feed‐forward loop that intertwines the DNA methylation regulator UHRF1 with homeobox genes via UMRs in ESCC. NKX2‐5 and LHX1 synergistically increase UHRF1 transcription. Consequently, UHRF1 recruits DNMT1, DNMT3A, NKX2‐5, and LHX1, fostering a reinforcing feed‐forward loop at the UMRs of the *NKX2‐5* and *LHX1* loci and thus increasing the expression of these oncogenic target genes. These findings shed valuable light on the mechanisms underlying transcriptional dysregulation in cancer and pave the way for the development of innovative epigenetic therapies for ESCC.

## 4 Experimental Section

### ESCC Multi‐Omics Data

A total of 150 pairs of primary ESCC tissues and their matched adjacent normal tissues were collected from Shanxi Medical University. Multi‐omics sequencing, which included WGS, WGBS, and RNA‐seq, was performed in accordance with the protocol described in the previous study.^[^ ^13^ ^]^ The data were deposited in the Genome Sequence Archive (GSA) in the BIG Data Center (http://bigd.big.ac.cn/gsa), Beijing Institute of Genomics (BIG), Chinese Academy of Sciences, with the BioProject number PRJCA004501.

### Public Data Resources

Pan‐cancer RNA‐seq data and somatic mutation information were downloaded from TCGA database (https://www.cancer.gov/tcga). The analysis included 24 cancer types for which normal tissue data were available. For expression data for various normal tissues, the study utilized the GTEx portal, which provides comprehensive data from more than 26 primary normal tissues derived from hundreds of healthy individuals.^[^ ^34^ ^]^ In addition, an independent RNA‐Seq dataset for ESCC was downloaded from the National Center for Biotechnology Information (NCBI) Sequence Read Archive (SRA) database under the accession code PRJNA665149.^[^ ^35^ ^]^ Furthermore, public human preimplantation development and esophagus development datasets were obtained from Yan et al. and Gao et al., respectively.^[^ ^36^, ^37^ ^]^

### Identification of Undermethylated Regions (UMRs) and Differentially Methylated Regions (DMRs)

For each WGBS profile, the study utilized trim_galore [https://github.com/FelixKrueger/TrimGalore] to eliminate adaptor sequences and low‐quality reads with the default threshold. After trimming, the remaining bisulfite‐treated reads were aligned to the reference human genome (hg38) using Bismark,^[^ ^38^ ^]^ which enabled to identify the methylation states at single‐base resolution. The PCR duplicates were then filtered out by using Picard (http://broadinstitute.github.io/picard/). To ensure the accuracy of CpG methylation detection, a coverage threshold of at least 4 reads was implemented.^[^ ^39^ ^]^ The CpG methylation information was extracted using Bismark's bismark_methylation_extractor module. A reference set of UMRs (refUMRs) was detected using the Hidden Markov Model within iBSTools (https://github.com/methylation/iBSTools) in normal samples. This resulted in the identification of a total of 21993 refUMRs. Differentially methylated regions (DMRs) were then identified using metilene (v.0.2‐8),^[^ ^16^ ^]^ with default criteria, including a minimum mean methylation difference of 0.1, a minimum of 10 CpGs, and an FDR of less than 0.05. In total, the analysis identified 8670 hypermethylated DMRs (hyper‐DMRs) and 250696 hypomethylated DMRs (hypo‐DMRs) for subsequent analysis. Furthermore, the DMR‐associated genes were annotated using the R package ChIPseeker.^[^ ^40^ ^]^ The list of CpG islands was downloaded from the UCSC genome browser (http://hgdownload.cse.ucsc.edu/goldenpath/hg38/database/cpgIslandExt.txt.gz). To visualize the methylation ratios across the transcription start site (TSS) and gene body, the study utilized DeepTools (v.3.1.3).^[^ ^41^ ^]^

### GSEA and Functional Enrichment Analysis

GSEA was conducted using the “clusterProfiler” R package, with the aim of pinpointing significantly enriched gene sets.^[^ ^42^ ^]^ The significance of the enrichment scores for each gene set was calculated using a permutation test with 1000 iterations. The false discovery rate (FDR) was calculated to adjust for multiple testing; gene sets with an FDR <0.05 were considered to be significantly enriched. The top enriched gene sets were subsequently identified and used for network construction. Furthermore, GO enrichment analysis and Kyoto Encyclopedia of Genes and Genomes (KEGG) pathway enrichment analysis were performed using DAVID (http://david.abcc.ncifcrf.gov/home.jsp) to identify significant terms enriched in upregulated and downregulated genes, respectively. Finally, terms and pathways were considered to be significantly enriched if they yielded an adjusted *P*‐value of <0.05.

### Establishment of the Gene Risk Score (GRS) Scoring System

For aHBGs, a gene risk score (*GRS_i_*) was developed that incorporates various types of multi‐omics information, including gene expression, DNA methylation, genetic mutation, and regulatory intensity. Specifically, *GRS_i_* is a weighted sum of several indicators for a given gene i, including the log_2_fold change (LFC), absolute differential methylation (DM) value, minor allele frequency (MAF), and regulatory network connectivity.

(1) $$\;GRS_{i}=\sum_{j=1}^{M}\rho_{\left(j,Z\right)}\;X_{ij}$$

where *X_ij_* is the *j*_*th*_ value of the LFC, absolute DM value, MAF, and regulatory network connectivity for gene *i*. The weight ρ_(*j*,*Z*)_ was determined by computing the Spearman correlation coefficient between factor *X_ij_* and the missense Z score of gene *i*. The missense Z score is a gene constraint metric used in gnomAD to assess missense variants.^[^ ^43^ ^]^ A gene with a missense Z score greater than or equal to 3.09 is considered intolerant to missense variation.

### Regulatory Network

The study conducted GSEA against a ranked list of aHBGs that were ranked by the *GRS_i_* score. The analysis revealed four significant gene sets: cell cycle, p53 signaling pathway, basal cell carcinoma, and hedgehog signaling pathway genes. Furthermore, the STRING database (version 11.0) was used with a combined score cutoff of ≥700 to determine the first‐order interactions between the four gene sets and the top 15 aHBGs.^[^ ^44^ ^]^ The resulting network was visualized using Cytoscape, and topological analysis was performed using the built‐in tool NetworkAnalyzer.^[^ ^45^ ^]^

### Cell Lines and Cell Culture

HEK293T cells were acquired from the American Type Culture Collection (ATCC, USA). The mouse ESCC cell line mEC25 was kindly provided by Dr. Li Fu from the Shenzhen University School of Medicine.^[^ ^46^ ^]^ Both HEK293T and mEC25 cells were cultured with DMEM containing 10% fetal bovine serum (FBS). The human KYSE ESCC cell lines KYSE30, KYSE150, KYSE180, KYSE450, KYSE410, and KYSE510 were kindly donated by Dr. Yutaka Shimada from Kyoto University. KYSE ESCC cells were grown in RPMI‐1640 medium supplemented with 10% FBS. The human ZEC ESCC cell lines ZEC014 and ZEC134 were established and generously provided by Dr. Dan Su from Zhejiang Cancer Hospital. They were cultured in DMEM/F12 supplemented with 10% FBS and nonessential amino acids (NEAA). The human esophageal epithelial cell lines NE2 and NE3 were provided by Dr. Enmin Li from the Medical College of Shantou University and were maintained in a mixture of K‐SFM (17005042, Gibco), EpiLife (MEPI500CA, Gibco) and EDGS (S0125, Gibco). All the cells were maintained in a humidified 5% CO_2_ atmosphere at 37 °C. The strains were regularly identified through short tandem repeat (STR) analysis and periodically detected for mycoplasma contamination to ensure their authenticity and sterility.

### Plasmids, Lentivirus Packaging, and Cell Screening

The coding sequences (CDSs) of the human *NKX2‐5* and *LHX1* genes were successfully inserted into the pLVX‐IRES‐Puro vector, while the CDSs of the human *UHRF1*, murine *NKX2‐5*, and *LHX1* genes were cloned and inserted into the pLVX‐IRES‐NEO vector. shRNA oligos targeting the *NKX2‐5*, *LHX1*, and *UHRF1* genes and nontarget sequences were constructed in the pSIH‐H1‐Puro vector. For the packaging of overexpressed lentivirus, 8×10^6^ HEK293T cells were seeded into 10‐cm cell culture dishes. Subsequently, 2.5 µg of pMD2. G (12259, Addgene), 7.5 µg of psPAX2 (12260, Addgene), and either 10 µg of pLVX‐IRES‐Puro‐hNKX2‐5/hLHX1 or pLVX‐IRES‐Neo‐hUHRF1/mNKX2‐5/mLHX1, along with their corresponding control plasmids, were cotransfected into HEK293T cells in 4 mL of Opti‐MEM and 70 µL of transfection reagent (40802ES03, Yeasen Biotechnology). For the packaging of the knockdown lentivirus, 2 µg of pMD2. G, 4 µg of psPAX2 and 8 µg of either pSIH‐H1‐Puro‐shNKX2‐5/shLHX1/shUHRF1 or their corresponding control plasmids were co‐transfected into HEK293T cells in 4 mL of Opti‐MEM and 60 µL of transfection reagent. Eight hours posttransfection, 6 mL of fresh medium was added to the culture dishes. After 48 hours, the lentivirus supernatant was harvested into a centrifuge tube, centrifuged at 3000 rpm for 20 min at 4 °C, and finally filtered through a 0.45‐µm syringe filter (Life Sciences). DNA methylation editing lentiviruses were purchased from Genechem (Shanghai, China). Subsequently, 2.5‐3×10^5^ ESCC cells were seeded into 6‐well culture plates and infected with lentiviruses for 48 hours. Stable cell lines were generated by selective screening with puromycin (1 µg mL^−1^, A610593, Sangon Biotech) or geneticin (400 µg mL^−1^, 11811031, Gibco) for one week. The shRNA sequences are available in Table S1 (Supporting Information).

### siRNA Transfection and Inhibitor Treatment

A mixture of 100 µL of Opti‐MEM medium, 6 µL of Lipofectamine 2000 (11668019, Invitrogen), and 100 nm siRNA or negative control was incubated for 20 min at room temperature. The mixture was subsequently added to 6‐well culture plates containing 3–4×10^5^ cells/well in 900 µL of Opti‐MEM. Twenty‐four hours later, the transfection medium was aspirated, and 2 mL of fresh medium was added to the culture dishes. The cells were then cultured continuously for 36 h. siRNAs targeting homeobox genes, UHRF1, and negative controls were purchased from Dharmacon, whereas siRNAs targeting DNMT1, DNMT3A, and negative controls were purchased from JTS Scientific. The siRNA sequences are available in Table S2 (Supporting Information). For inhibitor treatment, after the cell confluence reached 60%, the cells were treated with NSC232003 (HY‐103236, MCE) or its vehicle control for 24 h.

### RNA Isolation and Quantitative Real‐Time PCR

Total RNA was extracted from cultured cells using TRIzol and isopropyl alcohol. Subsequently, reverse transcription and quantitative real‐time PCR (qRT‒PCR) were performed via a QuantScript RT kit (KR116, TIANGEN) and PowerUp SYBR Green Master Mix (A25742, Applied Biosystems) following the manufacturer's instructions. β‐actin was used as a reference gene to normalize the expression of target genes, and the data were analyzed via the comparative Ct method. The primers used in this study are listed in Table S3 (Supporting Information).

### Western Blotting and Immunoprecipitation Experiments

The cells were washed with phosphate‐buffered saline (PBS) and lysed in RIPA lysis buffer (20101ES60, Yeasen Biotechnology) supplemented with protease inhibitor (Roche) for 20 min on ice. The lysates were subsequently treated with an ultrasonic processor for 40 s, followed by centrifugation at 12 000 rpm for 20 min at 4 °C. The protein concentration was determined with a BCA protein assay kit (23225, Thermo Fisher Scientific). Proteins (20–30 µg) were denatured with loading buffer at 99 °C for 10 min, separated by electrophoresis on a 10% SDS‒PAGE gel and transferred onto a PVDF membrane. The membranes were sequentially blocked with 5% skim milk at room temperature for 2 h, hybridized with the indicated antibodies (1:1000 dilution; LHX1, ab229474, Abcam; NKX2‐5, PA5‐49431, Invitrogen; UHRF1, 12387S, CST; 1:5000 dilution; β‐actin, A5316, Sigma) at 4 °C overnight, washed with TBST four times at room temperature, and incubated with the corresponding secondary antibodies (1:5000 dilution; HRP‐labeled goat anti‐rabbit IgG (H+L), ZB‐2306, ZSGB‐BIO; HRP‐labeled goat anti‐mouse IgG (H+L), ZB‐2305, ZSGB‐BIO) at room temperature for 2 h. After washing four times with TBST, the membranes were visualized using the SuperSignal West Femto Maximum Sensitivity Substrate Kit (34095, Thermo Fisher Scientific) and scanned using the ImageQuant LAS 4000 imaging system (GE Healthcare, USA). Immunoprecipitation experiments were performed in accordance with a previously described protocol.^[^ ^47^ ^]^

### Cell Proliferation Assay and Sphere Formation Assay

For the cell proliferation assays, KYSE30, KYSE180, KYSE150, KYSE450, KYSE510, and mEC25 cells were inoculated in 96‐well culture plates (2×10^3^‐3×10^3^ cells/well), with four duplicate wells in each group. After the cells had adhered to the plates, the culture medium was removed. A mixture of 10 µL of CCK‐8 reagent (C0005, TargetMoI) and 100 µL of serum‐free medium was added to each well and incubated at 37 °C for 1 h. The absorbance of each well was measured at 450 nm with a microplate reader at each time point (0, 2, and 4 days), and the growth curve of cell proliferation was calculated. For the colony formation assays, cells were seeded into 6‐well plates at a density of 1.5 × 10^3^–2 × 10^3^ cells/well, with three duplicate wells in each group. The culture medium was replaced with fresh medium every 3 days, and the colonies were cultured for 8–10 days. The colonies were fixed with methanol for 10 min and stained with 0.5% crystal violet for 6 h. After being adequately washed with water to remove excess dye, images of the colonies were taken, and the number of colonies was counted. For the sphere formation assay, cells were plated into the low‐attachment cell culture plates (24‐well) at a density of 1 × 10^3^ cells/plate in serum‐free DMEM/F12 containing 0.5% methylcellulose, 1% bovine serum albumin (BSA), 2% B27, 0.5 µg mL^−1^ insulin, 20 ng mL^−1^ EGF, and 20 ng mL^−1^ bFGF. The culture medium was supplemented with fresh medium every 2 days, and the spheres were cultured for 2–3 weeks. Finally, representative views were photographed, and the number of spheres was counted.

### RNA‐Seq

High‐quality total RNA was extracted via a RNeasy MinElute Cleanup Kit (74204, Qiagen). The cDNA libraries were constructed with a VAHTS Universal V6 RNA‐seq Library Prep Kit (NR604, Vazyme) and sequenced on the Illumina NovaSeq 6000 S4 platform (Mingma Technologies, China) to generate paired‐end reads of 2×150 bp. The raw RNA‐seq reads were then aligned to the human genome (hg38) using the STAR aligner with default settings.^[^ ^48^ ^]^ Gene‐level counts were obtained using htseq‐count,^[^ ^49^ ^]^ whereas transcript levels were quantified in terms of TPM and counted via RSEM.^[^ ^50^ ^]^ Lowly expressed genes were filtered out, and only genes that had a count >10 in at least 5% of the total 310 samples were retained. Ultimately, 27565 genes were retained for further analysis. Additionally, the samples underwent thorough quality control, during which any outliers with standardized sample network connectivity Z scores less than ‐2 were removed, as described in a previous protocol.^[^ ^51^ ^]^ A total of 300 samples were retained, including 150 ESCC samples and 150 matched adjacent normal samples. To identify differentially expressed genes between normal and tumor tissues, the DESeq2 R package was utilized.^[^ ^52^ ^]^ Genes were considered significantly differentially expressed if they had an absolute log2fold change (LFC) value of ≥1 and a false discovery rate (FDR) of ≤0.05.

### ChIP‐Seq

ChIP was performed as previously described.^[^ ^53^ ^]^ Briefly, cells and clipped tissues were crosslinked with 37% formaldehyde, which was terminated with glycine. Chromatins were fragmented with a micrococcal nuclease and ultrasonic processor and immunoprecipitated with the indicated antibodies (LHX1, sc‐515631X, Santa Cruz; NKX2‐5, PA5‐49431, Invitrogen; UHRF1, ab126243, Abcam; DNMT1, PA5‐30581, Invitrogen; DNMT3A, 49768S, CST) and ChIP‐grade protein G magnetic beads. After decrosslinking at 65 °C, the DNA fragments were purified with the SimpleChIP Plus Enzymatic Chromatin IP Kit (9005, CST). ChIP‐seq libraries were prepared using the NEBNext DNA Ultra II library prep kit (E7645L, New England Biolabs) and NEBNext Multiplex Oligos for Illumina (E7335S, New England Biolabs), and 2 × 150 bp were sequenced via Illumina NovaSeq. The raw ChIP‐seq reads were aligned to the human genome (hg38) using Bowtie2 (version 2.3.4).^[^ ^54^ ^]^ Quality control for the alignment BAM files was conducted using SAMtools,^[^ ^55^ ^]^ whereby only uniquely mapped reads were retained. Then, PCR duplicates were removed with Picard (http://broadinstitute.github.io/picard/) using the MarkDuplicates command with the parameters “VALIDATION_STRINGENCY = LENIENT REMOVE_DUPLICATES = true” for downstream analyses. Significant peaks for NKX2‐5 and LHX1 were identified via the MACS2 call peak tool with the default parameters except for the following: “‐f BAMPE ‐g hs ‐B –trackline ‐p 1e‐9 –nolambda”.^[^ ^56^, ^57^ ^]^ The peaks were annotated using the ChIPseeker package.^[^ ^40^ ^]^ Next, bigwig files were generated from the BAM files via deepTools,^[^ ^58^ ^]^ and the parameters were as follows: ‐binSize 50 ‐extendReads 200 and –normalize using RPKM. The signal intensities of each ChIP library against the corresponding input library were scored. The peak signal within a specific region was subsequently measured as the number of reads per kilobase per million (RPKM) via the UCSC tool bigWigAverageOverBed. Finally, the track files were visualized via the Integrated Genomics Viewer (IGV).^[^ ^59^ ^]^ The ChIP‒qPCR primers used are listed in Table S3 (Supporting Information).

### WGBS Sequencing

Genomic DNA (gDNA) was isolated from cells. A total of 200 ng of gDNA spiked with 1% unmethylated λDNA was randomly fragmented by sonication to 300 bp, followed by end repair and adenylation. The fragmented DNA was ligated with the methylated adapters, and then, bisulfite treatment was performed using the EZ DNA Methylation‐Gold Kit (D5005, Zymo). The DNA libraries were constructed with KAPA HiFi HotStart Uracil + ReadyMix (Roche). Paired‐end sequencing was performed using the Illumina NovaSeq 6000 S4 platform (Mingma Technologies, China) following Illumina‐provided protocols for 2 × 150 paired‐end sequencing.

### Tumor Xenograft Experiments

For the malignant transformation experiments, 100 µL of NE2 (1 × 10^6^ cells) or NE3 (1 × 10^6^ cells) cell suspensions were mixed with 100 µL of Matrigel, and then, 200 µL of each mixture was subcutaneously transplanted into 6‐week‐old male NCG mice (Gem‐Pharmatech Co., Ltd, Nanjing, China). Tumor formation was observed once a week, and the number of tumors was recorded. After 6 weeks, the mice were euthanized, and the tumors were dissected. For the transplantation experiments, 100 µL of KYSE30 (1 × 10^6^ cells) or KYSE450 (4 × 10^6^ cells) cell suspensions were implanted into 6‐week‐old male BALB/c nude mice, 100 µL of KYSE150 (8 × 10^5^ cells) cell suspensions were subcutaneously injected into 6‐week‐old female BALB/c nude mice, and 100 µL of mEC25 (3.5 × 10^6^ cells) cell suspensions mixed with 50 µL of Matrigel were subcutaneously transplanted into 6‐week‐old male C57BL/6J mice. For the inhibitor treatment experiments, KYSE450 cells were implanted into BALB/c nude mice. One week later, the mice were randomly divided into four groups that received vehicle, NSC232003 (target MOI, T12261, 5 mg kg^−1^), SGI‐1027 (target MOI, T1904, 5 mg kg^−1^) or a combination of NSC232003 and SGI‐1027. Inhibitors were administered intraperitoneally every two days for 3 weeks. The tumor size was measured every 3–4 days, and the tumors were harvested two‒three weeks after implantation. Finally, the tumors were photographed and weighed, and the tumor volumes were calculated according to the following formula: volume = (Length × Width^2^)/2. All animal studies were performed in accordance with animal ethical guidelines and approved by the Institutional Animal Care and Use Committee of the Chinese Academy of Medical Sciences Cancer Hospital (NCC2020A095).

### Statistical Analysis

Statistical analyses were performed, and the results were visualized using GraphPad Prism 9. Two‐tailed independent and paired Student's *t*‐tests were performed to determine statistical significance. The data are presented as the means ± SD, and differences were considered statistically significant at *P* < 0.05 unless otherwise specified. Pearson's correlation analysis was conducted to assess the associations between two genes and between two studies, whereas Spearman's correlation analysis was used to evaluate the associations between DNA methylation and gene expression. Venn diagram enrichment analysis was performed using hypergeometric tests. All tests in boxplots and violin plots were performed via the Wilcoxon test. Survival curves were drawn using the Kaplan‒Meier method, and survival analysis was conducted with the log‐rank test.

## Conflict of Interest

The authors declare no conflict of interest.

## Author Contributions

X.L., D.F., and Y.L. contributed equally to this work. J.S., Z.L., and H.C. conceptualized and supervised this project and edited the manuscript. X. L. and D.F. designed the experiments, performed the experiments and statistical analysis, and wrote the manuscript. Y.L. prepared the clinical samples. J.Y. and Q.Z. performed the bioinformatics analyses. W.S., X.W., and J.C. performed the animal experiments. L.Q., T.G., and N.Z. collected the pathological data of ESCC patients. All authors read and approved the final manuscript.

## Supporting information



Supporting Information

## Data Availability

The data that support the findings of this study are available from the corresponding author upon reasonable request.
