# Supplementary material for: NKX2‐5/LHX1 and UHRF1 Establishing a Positive Feedback Regulatory Circuitry Drives Esophageal Squamous Cell Carcinoma through Epigenetic Dysregulation
Source: Adv Sci (Weinh). 2025 Apr 30;12(20):2413508. doi: 10.1002/advs.202413508 (PMC12120717; doi:10.1002/advs.202413508)
Supplement: Supplementary file 1 — Supporting Information [file ADVS-12-2413508-s001.docx]

Supporting Information

**NKX2-5/LHX1 and UHRF1 establishing a positive feedback regulatory circuitry drives esophageal squamous cell carcinoma through epigenetic dysregulation**

*Xukun Li, Dandan Fan, Yong Li, Jian Yuan, Wanyuan Sun, Qinghao Zhu, Ling Qi, Xueling Wu, Jiahui Cai, Tongyang Gong, Ning Zhao,* *Jianzhong Su*^*^*, Zhihua Liu*^*^*, Hongyan Chen*^*^

**This PDF file includes:**

Supplementary Figures S1 to S7

Supplementary Tables S1 to S3

**Supplementary Fig S1.** **The genomic characteristics between aHBGs and uHBGs**. A) The boxplot revealing the difference in gene length, gene compactness, base and codon usage, gene essentiality and conservation between aHBGs and uHBGs. B) The boxplot showing the number of single nucleotide variants (SNVs) per 1kb between aHBGs and uHBGs in both TCGA pan-cancer and ESCC datasets. C, D) The somatic mutation of CpG dinucleotide in aHBGs and uHBGs in TCGA pan-cancer and ESCC datasets.

**Supplementary Fig S2. The correlation between gene body UMRs DNA hypermethylation and the overexpression of aHBGs**. A) The DNA methylation distribution across the gene bodies of all protein coding genes in ESCC tissues and paired adjacent normal tissues. B) Violin plots displaying the average methylation levels at promoter and gene body regions of hypermethylated aHBGs in ESCC cells (KYSE30, KYSE410, KYSE450, KYSE510, ZEC014) and normal esophageal epithelial cells (NE2, NE3). C) DNA methylation distribution across the gene bodies of aHBGs in ESCC cells and normal esophageal epithelial cells. D) Violin plots with embedded boxplots depicting the distribution of average methylation levels at UMRs in ESCC tissues and paired adjacent normal tissues.

**Supplementary Fig S3. aHBGs promoted the malignant transformation of normal esophageal epithelial cells**. A) Gene risk score for aHBGs in ESCC tissues by comprehensively analyzing the information of gene expression, DNA methylation, genetic mutation and regulatory intensity. The representative top aHBGs identified by Gene Risk Scoring system. B) The four significantly enriched gene sets identified by the GSEA analysis against the ranked list of aHBGs. C) Regulatory networks among the four interactomes from S3B and the top 15 aHBGs from S3A were generated with Cytoscape, with protein-protein interactions curated from the STRING database. The different gene sets’ classes were indicated using a color-coding scheme. D) The qRT-PCR analysis of DLX1, NKX2-5, LHX1, GBX2 and TLX3 expression in NE2 and NE3 cells. E) NKX2-5 and LHX1 protein levels in NE2 or NE3 cells infected with the NKX2-5 and LHX1 overexpression lentivirus or control lentivirus. F) Sphere formation assays were performed after the overexpression of NKX2-5 or LHX1 in normal esophageal epithelial NE2 or NE3 cells. Representative images of colonies (left) and statistical analyses of the number of spheres (right) are shown. Scale bars=50 or 100 μm.

**Supplementary Fig S4. Knockdown of NKX2-5 or LHX1 inhibited ESCC cell proliferation and tumor growth.** A, B) NKX2-5 and LHX1 mRNA and protein levels in KYSE450, KYSE510, KYSE150 and KYSE180 cells infected with the NKX2-5 and LHX1 shRNA lentivirus or control lentivirus. C, D) NKX2-5 and LHX1 mRNA and protein levels in mEC25 cells infected with the NKX2-5 and LHX1 overexpression lentivirus or control lentivirus. E, F) CCK-8 and colony formation assays were performed after the knockdown of NKX2-5 or LHX1 in KYSE510 and KYSE180 cells. Representative images of colonies (left) and statistical analyses of the number of colonies (right) are shown. G, H) Tumor weight of mice subcutaneously injected with NKX2-5-knockdown KYSE450, LHX1-knockdown KYSE150 cells and control cells (n=8). I, J) Tumor weight of mice subcutaneously transplanted with NKX2-5- and LHX1- overexpressing mEC25 cells and control cells (n=10). Data are presented as the mean ± SD, two-tailed *t*-tests, **P* < 0.05, ***P* < 0.01, ****P* < 0.001, *****P* < 0.0001.

**Supplementary Fig S5. NKX2-5 and LHX1 transcriptionally regulate the expression of UHRF1**. A) The distribution of NKX2-5- and LHX1 binding sites in ESCC tissues and paired adjacent normal tissues. B, C) KYSE450 and KYSE180 were transfected with NKX2-5/LHX1 siRNA and control siRNA. The knockdown efficiency of NKX2-5 and LHX1 were detected by qRT-PCR. D) Integrative genomic viewer tracks showing normalized ChIP-seq of NKX2-5, LHX1 and other histone marker antibodies surrounding *UHRF1* loci. E) The enrichment of NKX2-5 and LHX1 at the region of *UHRF1* loci in KYSE450 and KYSE180 cells, the binding sites of NKX2-5 and LHX1 on the promoter of UHRF1 located at chr19: 4911559-4911883 (NKX2-5) and chr19: 4911375-4911880 (LHX1). F, G) UHRF1 mRNA and protein levels in KYSE30, KYSE450 and KYSE510 cells infected with the UHRF1 shRNA lentivirus or control lentivirus. H) Tumor weight of mice subcutaneously inoculated with UHRF1-knockdown KYSE30 and control cells (n=10). I, J) NKX2-5, LHX1 and UHRF1 mRNA and protein levels in KYSE450 cells featuring knockdown of NKX2-5 and LHX1 genes, coupled with exogenous overexpression of UHRF1. K) Tumor weight of mice subcutaneously injected with NKX2-5- and LHX1- knockdown KYSE450 cells plus the UHRF1overexpression and control cells (n=7). Data are presented as the mean ± SD, two-tailed *t*-tests, **P* < 0.05, ***P* < 0.01, ****P* < 0.001, *****P* < 0.0001.

**Supplementary Fig S6. UHRF1-mediated DNA methylation upregulated the expression of NKX2-5 and LHX1**. A) DNA methylation levels at gene bodies of *NKX2-5* and *LHX1* loci in ESCC tissues and adjacent normal tissues, as well as ESCC cells and normal esophageal epithelial cells. B) Average methylation levels of UHRF1-knockdown KYSE450 and control cells at gene bodies of *Homeobox gene* loci. C) Methylation levels of UHRF1-knockdwon KYSE450 and control cells at gene bodies of *NKX2-5* and *LHX1* loci. D) Co-Immunoprecipitation assays showing the interaction changes between UHRF1 and DNMT1/DNMT3A in KYSE450 cells after UHRF1 knockdown. E) The qRT-PCR analysis of UHRF1 mRNA expression in KYSE450 cells transfected with UHRF1 siRNA or control siRNA. F) The qRT-PCR analysis of UHRF1, NKX2-5 and LHX1 mRNA expression in KYSE450 cells infected with UHRF1 shRNA lentivirus or control lentivirus. G, H) The expression of NKX2-5 and LHX1 in KYSE510 cells transfected with DNMT1, DNMT3A siRNA or control siRNA was detected by qRT-PCR. I) The qRT-PCR analysis showing the NKX2-5 and LHX1 expression in KYSE180 cells treated with or without UHRF1 inhibitor NSC232003. J) The qRT-PCR analysis of endogenous NKX2-5 and LHX1 mRNA expression in KYSE450 cells transfected with pLVX-puro-NKX2-5/LHX1 plasmids or control plasmids. K) Co-Immunoprecipitation assays showing the interaction changes between UHRF1 and NKX2-5/LHX1/DNMT1/DNMT3A in KYSE450 cells. Data are presented as the mean ± SD, two-tailed *t*-tests, **P* < 0.05, ***P* < 0.01, ****P* < 0.001, ns means no significance.

**Supplementary Fig S7. Prognostic and therapeutic potential of NKX2-5/LHX1/UHRF1 in ESCC**. A) Spearman’s correlation between UHRF1 mRNA expression and NKX2-5 and LHX1 mRNA expression in ESCC tissues and adjacent normal tissues. B) Tumor weight of mice injected with KYSE450 cells under the administration of vehicle, UHRF1 inhibitor, DNMTs inhibitor or combined treatment (n=7). Data are presented as the mean ± SD, two-tailed *t*-tests, *****P* < 0.0001, ns means no significance.

| **Supplementary Table S1. shRNAs and sgRNAs used in this study.** | | |
| --- | --- | --- |
| Name | Sequence (5' to 3') | Species |
| shNC-F | GATCCTGGTTTACATGTTTTCTGACTTCCTGTCAGATCAGAAAACATGTAAACCATTTTTG | Human |
| shNC-R | AATTCAAAAATGGTTTACATGTTTTCTGATCTGACAGGAAGTCAGAAAACATGTAAACCAG | Human |
| shLHX1#2-F | GATCCGCAAAGAGGATTACCTAAGCTTCCTGTCAGACTTAGGTAATCCTCTTTGCTTTTTG | Human |
| shLHX1#2-R | AATTCAAAAAGCAAAGAGGATTACCTAAGTCTGACAGGAAGCTTAGGTAATCCTCTTTGCG | Human |
| shLHX1#3-F | GATCCGGCAAACTCTACTGCAAGACTTCCTGTCAGATCTTGCAGTAGAGTTTGCCTTTTTG | Human |
| shLHX1#3-R | AATTCAAAAAGGCAAACTCTACTGCAAGATCTGACAGGAAGTCTTGCAGTAGAGTTTGCCG | Human |
| shNKX2-5#3-F | GATCCCAAGTGTGCGTCTGCCTTTCTTCCTGTCAGAAAAGGCAGACGCACACTTGTTTTTG | Human |
| shNKX2-5#3-R | AATTCAAAAACAAGTGTGCGTCTGCCTTTTCTGACAGGAAGAAAGGCAGACGCACACTTGG | Human |
| shNKX2-5#4-F | GATCCGGGATTCCGCAGAGCAACTCTTCCTGTCAGAAGTTGCTCTGCGGAATCCCTTTTTG | Human |
| shNKX2-5#4-R | AATTCAAAAAGGGATTCCGCAGAGCAACTTCTGACAGGAAGAGTTGCTCTGCGGAATCCCG | Human |
| shUHRF1#5-F | GATCCGCGGAACAGTCTTGTGATCCTTCCTGTCCAGAGATCACAAGACTGTTCCGCTTTTTG | Human |
| shUHRF1#5-R | AATTCAAAAAGCGGAACAGTCTTGTGATC TCTGACAGGAAG GATCACAAGACTGTTCCGCG | Human |
| shUHRF1#6-F | GATCCGCCATACCCTCTTCGACTACTTCCTGTCAGATAGTCGAAGAGGGTATGGCTTTTTG | Human |
| shUHRF1#6-R | AATTCAAAAAGCCATACCCTCTTCGACTA TCTGACAGGAAG TAGTCGAAGAGGGTATGGCG | Human |

| **Supplementary Table S2. siRNAs used in this study.** | | | |
| --- | --- | --- | --- |
| Name | Sequence (5' to 3') or Catalog | Species | Company |
| ON-TARGETplus Control siRNA | UGGUUUACAUGUCGACUAA | Human | Dharmacon |
|  | UGGUUUACAUGUUGUGUGA |  |  |
|  | UGGUUUACAUGUUUUCUGA |  |  |
|  | UGGUUUACAUGUUUUCCUA |  |  |
| DLX1 | AAACGAAUAAGGAGGACGU | Human | Dharmacon |
|  | UGGAGAGUUUGGAGCCGAA |  |  |
|  | CAUCCAUCCCGUCCGGAAA |  |  |
|  | GGGUAGAAAGAGGGAGCGA |  |  |
| PAX6 | CCAAGCGUGUCAUCAAUAA | Human | Dharmacon |
|  | GGCAAUCGGUGGUAGUAAA |  |  |
|  | GUGCGACAUUUCCCGAAUU |  |  |
|  | AACCUGAUAUGUCUCAAUA |  |  |
| GBX2 | GGAAAGACGAGUCAAAGGU | Human | Dharmacon |
|  | GGACUGCCUUCACCAGCGA |  |  |
|  | GCGCUCACCUCUACGCUCA |  |  |
|  | UAGCACCGCCUUCAGCAUA |  |  |
| EN2 | GCAAGUCGGACAGCGAGUA | Human | Dharmacon |
|  | GGACCGGCCUUCUUCAGGU |  |  |
|  | GAACAAAGAGGACAAGCGG |  |  |
|  | GCGCUAAACAAUGCAAUAA |  |  |
| LHX2 | GCGCUAAGCUGCAACGAAA | Human | Dharmacon |
|  | GCCAGAAGACCAAGCGCAU |  |  |
|  | CGGCCGAGGAAACGUAAGA |  |  |
|  | GCGCUCGGGACUUGGUUUA |  |  |
| NKX2-1 | AGGCCAAACUGCUGGACGU | Human | Dharmacon |
|  | GCAAAGAGGACUCGCUUGU |  |  |
|  | GCUGUAAAACGCUGUCAAA |  |  |
|  | CGCGAAAAUAGUUUGUUUA |  |  |
| HMX1 | GGGCGUUAGGGAAGGGAUG | Human | Dharmacon |
|  | CCGGUGCUCUACCACGAAA |  |  |
|  | GAGCGCCUCUAGAAUGUAA |  |  |
|  | GAAUCUACUUAUUUGCGUA |  |  |
| LHX1 | GAGAUUACCAGAGCGAGUA | Human | Dharmacon |
|  | GCAAAGAGGAUUACCUAAG |  |  |
|  | GGCAAACUCUACUGCAAGA |  |  |
|  | GCAACGAGAAUGACGACCA |  |  |
| NKX2-5 | CCUCAAUCCCUACGGUUAU | Human | Dharmacon |
|  | ACAACAACUUCGUGAACUU |  |  |
|  | CAAGUGUGCGUCUGCCUUU |  |  |
|  | GGGAUUCCGCAGAGCAACU |  |  |
| CDX2 | ACAAAUAUCGAGUGGUGUA | Human | Dharmacon |
|  | CUACGGCGGUUACCACGUG |  |  |
|  | UCACUACAGUCGCUACAUC |  |  |
|  | GACGUGAGCAUGUACCCUA |  |  |
| VAX1 | GUUCGGGAAACCAGACAAA | Human | Dharmacon |
|  | GAGAGAUCAUCCUGCCCAA |  |  |
|  | CCUAAGAGGACGCGCACGU |  |  |
|  | GCGCUGCUGAGGAUUGUAA |  |  |
| POU4F1 | UGAAAUUCUCUGCCACUUA | Human | Dharmacon |
|  | CCGAGAAACUGGACCUCAA |  |  |
|  | GAACAAGCCUGAGCUCUUC |  |  |
|  | UCGUAGAGAUGGUUUGUUA |  |  |
| TLX3 | UGCCCAAACGUGUAAAUAA | Human | Dharmacon |
|  | GCGGGAUCUUACAGUGUGA |  |  |
|  | GGAGGAUAGUUCCAAGGUU |  |  |
|  | CAAGAAAGCGCCUUACGUU |  |  |
| UHRF1 | GCGGAACAGUCUUGUGAUC | Human | Dharmacon |
|  | GCCAUACCCUCUUCGACUA |  |  |
|  | GCAAGGGCAUGGCCUGUGU |  |  |
|  | GUAAAGUGGAGGAGACGUU |  |  |
| DNMT1 | GCUUCAAUUCGCGCACCUATT | Human | JTS |
|  | UAGGUGCGCGAAUUGAAGCTT |  |  |
| DNMT3A | CCACCAGAAGAAGAGAAGATT | Human | JTS |
|  | UCUUCUCUUCUUCUGGUGGTT |  |  |

| **Supplementary Table S3. Primers used in this study.** | | | |
| --- | --- | --- | --- |
| Name | Sequence (5' to 3') | Species |  |
| hLHX1-F | GCCAAAGAGAACAGCCTTCACTC | Human | qPCR |
| hLHX1-R | GGTCGTCATTCTCGTTGCTACC | Human | qPCR |
| hNKX2-5-F | CAAGTGTGCGTCTGCCTTTC | Human | qPCR |
| hNKX2-5-R | CGCACAGCTCTTTCTTTTCGG | Human | qPCR |
| hUHRF1-F | GACAAGCAGCTCATGTGCGATG | Human | qPCR |
| hUHRF1-R | AGTACCACCTCGCTGGCATCAT | Human | qPCR |
| hβ-actin-F | AGGCACCAGGGCGTGAT | Human | qPCR |
| hβ-actin-R | GCCCACATAGGAATCCTTCTGAC | Human | qPCR |
| mLHX1-F | CCCATCCTGGACCGTTTCC | Mouse | qPCR |
| mLHX1-R | CGCTTGGAGAGATGCCCTG | Mouse | qPCR |
| mNKX2-5-F | GACAAAGCCGAGACGGATGG | Mouse | qPCR |
| mNKX2-5-R | CTGTCGCTTGCACTTGTAGC | Mouse | qPCR |
| mβ-actin-F | CATTGCTGACAGGATGCAGAAGG | Mouse | qPCR |
| mβ-actin-R | TGCTGGAAGGTGGACAGTGAGG | Mouse | qPCR |
| hDNMT1-F | AGGTGGAGAGTTATGACGAGGC | Human | qPCR |
| hDNMT1-R | GGTAGAATGCCTGATGGTCTGC | Human | qPCR |
| hDNMT3A-F | CCTCTTCGTTGGAGGAATGTGC | Human | qPCR |
| hDNMT3A-R | GTTTCCGCACATGAGCACCTCA | Human | qPCR |
| hGBX2-F | CAGGCTTCGCTCGTCGG | Human | qPCR |
| hGBX2-R | CTGTAGTCCACATCGCTCTCC | Human | qPCR |
| hDXL1-F | CATCAGTTCGGTGCAGTCCTAC | Human | qPCR |
| hDXL1-R | CCTTGCCATTGAAGCGCACTTC | Human | qPCR |
| hTXL3-F | GAGGACGCGGGATCTTACAG | Human | qPCR |
| hTXL3-R | TGTGAAGCGGTCTTTCACGA | Human | qPCR |
| NKX2-5-F for endogenous | CAACATGACCCTGAGTCCCC | Human | qPCR |
| NKX2-5-R for endogenous | TAATCGCCGCCACAAACTCT | Human | qPCR |
| LHX1-F for endogenous | GAGACCGGATGGAAAAGGGG | Human | qPCR |
| LHX1-R for endogenous | GAGACCGGATGGAAAAGGGG | Human | qPCR |
| NKX2-5 ChIP-F | CGTAGGCCTCTGGCTTGAA | Human | ChIP-qPCR |
| NKX2-5 ChIP-R | CCACGCCCTTCTCAGTCAAA | Human | ChIP-qPCR |
| LHX1 ChIP-F | CGGATCAGAGGTTAGCGTGG | Human | ChIP-qPCR |
| LHX1 ChIP-R | CAGCCAAACCCAGACCCATA | Human | ChIP-qPCR |
| UHRF1-ChIP-F | ATTTCATAGCTGCCACGTTTGGG | Human | ChIP-qPCR |
| UHRF1-ChIP-R | CACCTTCGATCACACTGTCTCAT | Human | ChIP-qPCR |
